# Fracture-related outcome study for operatively treated tibia shaft fractures (F.R.O.S.T.): registry rationale and design

**DOI:** 10.1186/s12891-020-03930-x

**Published:** 2021-01-09

**Authors:** Willem-Jan Metsemakers, Kirsten Kortram, Nando Ferreira, Mario Morgenstern, Alexander Joeris, Hans-Christoph Pape, Christian Kammerlander, Sanjit Konda, Jong-Keon Oh, Peter V. Giannoudis, Kenneth A. Egol, William T. Obremskey, Michael H. J. Verhofstad, Michael Raschke, on behalf of the F.R.O.S.T. Study Group

**Affiliations:** 1 Department of Trauma Surgery, University Hospitals Leuven, Leuven, Belgium; 2 Department of Development and Regeneration, KU Leuven, Leuven, Belgium; 3 Trauma Research Unit, Department of Trauma Surgery, Erasmus MC, University Medical Center Rotterdam, Rotterdam, The Netherlands; 4 Division of Orthopaedics, Faculty of Medicine and Health Sciences, Stellenbosch University, Tygerberg Hospital, Cape Town, South Africa; 5 Department of Orthopaedic and Trauma Surgery, University Hospital Basel, Basel, Switzerland; 6 AO Innovation Translation Center, AO Foundation, Dübendorf, Switzerland; 7 Department of Trauma, UniversitätsSpital Zürich, University of Zurich, Raemistrasse, Zurich, Switzerland; 8 Department of General Trauma and Reconstructive Surgery, University Hospital, LMU Munich, Munich, Germany; 9 Department of Orthopaedic Surgery, NYU Langone Orthopedic Hospital and Jamaica Hospital Medical Center, New York, NY USA; 10 Department of Orthopaedic Surgery, Korea University College of Medicine, Guro Hospital, Guro-gu, Seoul, Republic of Korea; 11 Academic Department of Trauma and Orthopaedics, School of Medicine, University of Leeds, Leeds General Infirmary, Leeds, UK; 12 NIHR Leeds Biomedical Research Center, Chapel Allerton Hospital, Leeds, UK; 13 Department of Orthopedic Surgery, NYU Langone Orthopedic Hospital, New York, NY USA; 14 Department of Orthopaedic Surgery and Rehabilitation, Vanderbilt University Medical Center, Nashville, TN USA; 15 Department of Trauma-, Hand- and Reconstructive Surgery, University Hospital Muenster, Muenster, Germany

**Keywords:** Fracture-related infection, Tibial shaft fracture, Fracture fixation, Complications, Nonunion, Fracture, Infection, Registry

## Abstract

**Background:**

Tibial shaft fractures (TSFs) are among the most common long bone injuries often resulting from high-energy trauma. To date, musculoskeletal complications such as fracture-related infection (FRI) and compromised fracture healing following fracture fixation of these injuries are still prevalent. The relatively high complication rates prove that, despite advances in modern fracture care, the management of TSFs remains a challenge even in the hands of experienced surgeons. Therefore, the Fracture-Related Outcome Study for operatively treated Tibia shaft fractures (F.R.O.S.T.) aims at creating a registry that enables data mining to gather detailed information to support future clinical decision-making regarding the management of TSF’s.

**Methods:**

This prospective, international, multicenter, observational registry for TSFs was recently developed. Recruitment started in 2019 and is planned to take 36 months, seeking to enroll a minimum of 1000 patients. The study protocol does not influence the clinical decision-making procedure, implant choice, or surgical/imaging techniques; these are being performed as per local hospital standard of care. Data collected in this registry include injury specifics, treatment details, clinical outcomes (e.g., FRI), patient-reported outcomes, and procedure- or implant-related adverse events. The minimum follow up is 12 months.

**Discussion:**

Although over the past decades, multiple high-quality studies have addressed individual research questions related to the outcome of TSFs, knowledge gaps remain. The scarcity of data calls for an international high-quality, population-based registry. Creating such a database could optimize strategies intended to prevent severe musculoskeletal complications. The main purpose of the F.R.O.S.T registry is to evaluate the association between different treatment strategies and patient outcomes. It will address not only operative techniques and implant materials but also perioperative preventive measures. For the first time, data concerning systemic perioperative antibiotic prophylaxis, the influence of local antimicrobials, and timing of soft-tissue coverage will be collected at an international level and correlated with standardized outcome measures in a large prospective, multicenter, observational registry for global accessibility.

**Trial registration:**

ClinicalTrials.gov: NCT03598530.

**Supplementary Information:**

The online version contains supplementary material available at 10.1186/s12891-020-03930-x.

## Background

Tibial shaft fractures (TSFs) are among the most common long bone injuries [1]. Their incidence has been reported on several occasions and varies between 8.1 and 37.0 per 100,000 persons/year, with a decrease over recent years [1–3]. Over 15% of tibia fractures are classified as open, representing the most common open long bone fracture [4, 5]. This is one of the reasons why musculoskeletal complications such as fracture-related infection (FRI) and compromised fracture healing remain prevalent with these types of injuries. Currently, the incidence of FRI ranges from 1%, following operative fixation of closed low-energy fractures [6], to 25–30% in complex open tibia fractures [5]. Nonunion rates vary from 3 to 17% [7–9]. Furthermore, annual reoperation rates following the operative treatment of TSFs have been reported to vary between 12 and 44% [10]. These high complication rates suggest that despite advances in modern fracture care, treatment of TSFs poses a challenge even for experienced surgeons.

Although several high-quality studies have already addressed individual research questions, knowledge gaps remain. Hence, the aim of the Fracture-Related Outcome Study for operatively treated Tibia shaft fractures (F.R.O.S.T.) is to create a registry that allows data mining to gather information that will assist in future clinical decision-making.

## Methods/design

### Study objectives

The registry’s main objective is to collect data on the operative treatment and outcome of TSFs in patients aged ≥18 years in an international and multicenter setting.

Specifically, this registry aims to:

- Increase knowledge and evidence with respect to the epidemiology and treatment concepts of TSFs worldwide by collecting prospective data in a structured and systematic manner.
- Identify risk factors (e.g., injury, patient, or treatment) for favorable and unfavorable outcomes and complications.
- Allow data mining to gather evidence to optimize the clinical decision-making process, particularly to (i) develop strategies that intend to prevent musculoskeletal complications and (ii) generate hypotheses for future studies.

### Study design

This is a prospective, international, multicenter, observational registry. Patient treatment is being performed according to the local standard of care (routine), that is, the study protocol (supplementary material) does not influence the clinical decision-making procedure or materials or surgical/imaging techniques. The sites that are currently included are summarized in Table 1.

**Table 1:** Sites currently included in the Fracture-Related Outcome Study for operatively treated Tibia shaft fractures (F.R.O.S.T.)

**Department of Trauma Surgery, University Hospitals Leuven, Leuven, Belgium**	**PI (PCI):** Willem-Jan Metsemakers
**Department of Trauma-, Hand- and Reconstructive Surgery, University Hospital Muenster, Muenster, Germany**	**PI (Co-PCI):** Michael Raschke
**Department of Trauma Surgery, Erasmus MC, University Medical Center Rotterdam, Rotterdam, The Netherlands**	**PI (Co-PCI):** Michael H. J. Verhofstad
**Department of Orthopaedic and Trauma Surgery, University Hospital Basel, Basel, Switzerland**	**PI** Mario Morgenstern
**Division of Orthopaedics, Faculty of Medicine and Health Sciences, Stellenbosch University, Tygerberg Hospital, Cape Town, South Africa**	**PI** Nando Ferreira
**Department of General Trauma and Reconstructive Surgery, University Hospital, LMU Munich, Munich, Germany**	**PI** Christian Kammerlander
**Department of Orthopaedic Surgery and Rehabilitation, Vanderbilt University Medical Center, Nashville, TN, United States of America**	**PI** William T. Obremskey
**Department of Orthopedic Surgery, NYU Langone Orthopedic Hospital, New York, NY, United States of America**	**PI** Kenneth A. Egol
**Department of Orthopaedic Surgery, NYU Langone Orthopedic Hospital and Jamaica Hospital Medical Center, New York, NY, United States of America**	**PI** Sanjit Konda
**Department of Orthopaedic Surgery, Korea University College of Medicine, Guro Hospital, Guro-gu, Seoul, Republic of Korea**	**PI** Jong-Keon Oh
**Department of Orthopaedic Surgery, Sengkang General Hospital, Singapore**	**PI** Wong Merng Koon
**Department of Trauma, UniversitätsSpital Zürich, University of Zurich, Zurich, Switzerland**	**PI** Hans-Christoph Pape
**Academic Department of Trauma and Orthopaedics, School of Medicine, University of Leeds, Leeds General Infirmary, Leeds, United Kingdom** ***(Planned New Site)***	**PI** Peter V. Giannoudis
**Department of Orthopaedics and Traumatology, University Medical Center, Johannes Gutenberg-University, Mainz, Germany** ***(Planned New Site)***	**PI** Eric Hanke
**Department of Orthopaedics and Traumatology, Queen Mary Hospital, University of Hong Kong, Pokfulam, Hong Kong** ***(Planned New Site)***	**PI** Christian Fang
**Department of Surgery, Franciscus Gasthuis en Vlietland Hospital, Rotterdam, The Netherlands** ***(Planned New Site)***	**PI** Taco M. A. L. Klem
**Department of Orthopedics, Elisabeth-TweeSteden Hospital, Tilburg, Netherlands** ***(Planned New Site)***	**PI** Kirsten Kortram

Site selection was based on a world-wide open call by the AO Foundation. It was decided that sites should be included from different parts of the world. The following criteria were developed during the decision-making process:

1. A site should be able to recruit a minimum number of patients within the planned recruitment time (a minimum of 65–75 patients per year).

2. A site should make sure that patients are in follow up for a minimum time of 1 year.

3. Sites must be able to anonymize their clinical data (e.g., radiological images).

4. Sites are able to plan patient visits as per standard of care at the site.

5. Sites should agree to the administrative conditions (reimbursement, contracts) and receive approval from their local ethical committee.

6. Sites should be able to perform registry related documentation

*PCI* Principal Coordinating Investigator, *co-PCI* co-Principal Coordinating Investigator, *PI* local Principal Investigator

### Participants

#### Sample size estimation

**Fig. 1:**
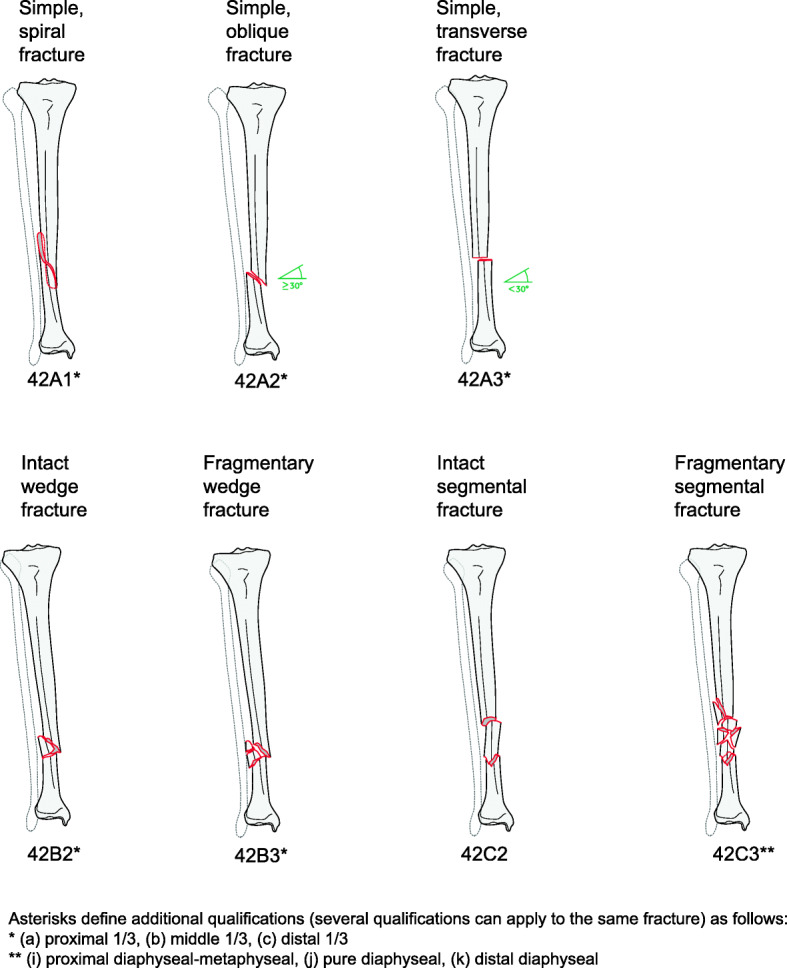
AO/OTA tibial shaft fracture (TSF) classification

Owing to the exploratory nature of this registry, there is no formal sample size calculation. Approximately 1000 tibial shaft fractures are expected to be included in the 3 years enrollment period. A sample size of 1000 will allow the identification of infrequent adverse events (AEs) as well as rare treatment concepts. The following inclusion and exclusion criteria are specified:

- Inclusion criteria
  1. Aged ≥18 years at the time of the injury.
  2. The diagnosis of a primary TSF, a fracture type 42, according to the Arbeitsgemeinschaft für Osteosynthesefragen (AO)/Orthopaedic Trauma Association (OTA) Fracture and Dislocation Classification (Fig. 1), that will be treated operatively as part of the standard of care.
  3. Informed consent will be obtained based on:
    - The ability of the patient or an assigned representative to understand the content of the patient information/Informed Consent Form (ICF).
    - Signed and dated Institutional Review Board (IRB)/Ethics Committee (EC)-approved written informed consent.
- Exclusion criteria
  1. Pathological fractures caused by malignancy.
  2. Patients participating in any other medical device or medicinal product study within the previous month that could influence the results of the present study.
  3. Patients who cannot provide independent written informed consent unless defined, and IRIR/EC-approved procedures for consenting such vulnerable patients are in place.

### Procedures

#### Recruitment

First, the investigator or another appropriately trained member of the research team at the study site identifies all eligible patients. These patients should meet all inclusion criteria and none of the exclusion criteria. Next, they inquire about the patient’s interest in participating in this study. If the patient wishes to participate, a legally eligible member of the research team goes through the informed consent process with the patient, explaining the study’s purpose, procedures, risk/benefits, alternatives to participation, and data protection. Each patient who chooses to participate signs and dates an ICF*.* All consenting patients are allocated a unique patient trial number. A protocol deviation is documented for patients found ineligible after treatment, indicating which inclusion/exclusion criterion (or criteria) was (or were) violated.

The follow-up (FU) population will consist of patients who have signed informed consent, are eligible (i.e., meet all inclusion and none of the exclusion criteria), and have commenced treatment (i.e., definitive treatment). Recruitment is planned to take 36 months to enroll a minimum of 1000 patients. An evaluation will take place at the end of this period. If deemed necessary, due to a high percentage of dropouts for example, the study period may be extended.

#### Baseline parameters

During their first visit to the emergency department or urgent care center, patients are assessed for eligibility. If informed consent is obtained, parameters regarding demographics, medical history, and injury are collected. Radiographic images and/or clinical pictures are taken as per local standard of care (routine) procedures. Demographic data, such as age, sex, height, weight, and ethnicity, are recorded. Medical history is documented in sufficient detail to calculate the Charlson Comorbidity Index, which has been designed and validated to classify prognostic comorbidity in longitudinal studies [11, 12]. Its prognostic accuracy has also been validated for a combined age-comorbidity index [13]. The recorded information includes the history of cardiovascular diseases, asthma, chronic lung disease, diabetes, renal disease, chronic liver disease, gastric or peptic ulcers, cancer, dementia, rheumatic or connective tissue disease, immunocompromised status [human immunodeficiency virus and/or others], and American Society of Anesthesiologists scores.

Information on medication history comprises the use of antibiotics, osteoporosis treatment, chronic use of analgesics and/or corticosteroids, and alcohol and drug intake. Information concerning the injury includes which side of the body the fracture is located on, high- or low-energy trauma, injury severity score, and fracture classification according to the AO/OTA fracture and dislocation classification [14]. Detailed information on additional fractures of the lower extremities both ipsilateral and contralateral will be collected. Furthermore, details of additional injuries will also be collected (e.g. cerebral, spine, thorax, abdomen, pelvis, upper extremity). Soft-tissue injury is to be classified according to Tscherne [15] for closed fractures and according to Gustilo-Anderson [16] and the OTA Open Fracture Classification [17] for open fractures. Concomitant fractures of the tibia or fibula are to be classified according to the AO/OTA fracture and dislocation classification [14].

#### Interventions and study procedures

Patients will undergo fracture fixation using osteosynthesis, including single- or multiple-staged procedures (e.g., initial external fixation with later conversion to internal fixation). The operative treatment for TSFs in adults mainly depends on the fracture characteristics and extent of the soft tissue damage and is to be performed according to the standard (routine) care procedures based on the individual clinician’s judgment and patient characteristics. Neither specific treatment nor specific time points for FU visits are dictated for registry purposes.

Registry-related assessments will include radiographic evaluations, performed by the treating surgeon at the treatment visit and at defined time points after treatment, following each site’s standard (routine) visit schedule for up to 36 months. Radiographic images and/or clinical pictures will be stored to enable future ad-hoc analysis after de-identification. If patients are unable to attend the last planned FU visit, they will be contacted by e-mail or phone to document the final outcomes, especially patient-reported outcomes (PROs) and complications not previously captured. The planned registry schedule is displayed in Table 2 over the 36-months FU visit period.

**Table 2:** Registry schedule for the Fracture-Related Outcome Study for operatively treated Tibia shaft fractures (F.R.O.S.T.)

	Baseline	Treatment	Post-treatment visit 1^a^	Post-treatment visit 2^a^	Post-treatment visit 3^a^	Additional Post-treatment visit(s)^a^
	–	Day 0	6 weeks (target 42 days: range 14 to 105)	6 months (target 183 days; range 106 to 260)	12 months (target 365 days, range 261 to 456)	Up to 36 months
Eligibility	X					
Patient information/consent	X^b^					
Demographics	X					
Medical history and pre-treatment values	X					
Injury(s) details	X					
Treatment details		X^c^				
Clinical outcome(s)			X	X	X	X
Patient-reported outcome(s)						
PROMIS: Global Health	X		X	X	X	X
PROMIS: Physical Function	X		X	X	X	X
PROMIS: Pain Interference			X	X	X	X
Pain numeric rating scale (NRS)			X	X	X	X
Complications			X	X	X	X
Images and/or other clinical pictures	X^d^	X^d^	X^d^	X^d^	X^d^	X^d^

^a^ If a patient does not visit at the specified time point, the visits will be assigned according to the specified rules. Additional postoperative visits may take place up to 36 months after day 0

^b^ Informed consent may occur after treatment under certain circumstances (see details in section 10). No data can be collected in the eCRF prior to consent

^c^ Treatment may occur on the same day as the baseline. Treatment of tibial fractures may require two or more stage-procedures (e.g., external fixation and secondary internal fixation) performed on different days

^d^ Images (X-ray images, CT scans, etc.) and clinical pictures are collected ONLY if they are taken as part of the standard of care (routine) procedures. All images and photographs will be de-identified

### Outcome measures

The data collected in this registry will include treatment details, clinical outcomes, PROs, and procedure- or implant-related AEs. Documentation of treatment details will comprise information about the time from injury to index surgery, duration of surgery, number of stages of the procedure, skin preparation, tourniquet use, details about debridement and irrigation, type of reduction/fixation method, specifics related to the fixation devices (e.g., material type, implant dimension*)*, intraoperative blood loss, as well as the use of local agents to promote bone healing. Moreover, information relating to the perioperative use of either prophylactic or curative systemic antibiotics and application of local antimicrobials will be documented and entered in the database. Furthermore, information pertaining to soft-tissue management will be recorded, such as timing and details of wound closure, soft-tissue coverage, and flap type. For concomitant fractures of the tibia or fibula, the treatment method will be documented. If further treatments are performed during the index surgery, these will also be captured.

Clinical outcome documentation will include time to bone healing/union, the presence of malalignment, malrotation or malunion, and the time to full weight bearing. Time to bone healing is defined as the time elapsed between definitive treatment and bony union. The healing status assessment will be based on a combination of clinical and radiological parameters, which will be judged by the treating surgeon. Clinically, healing will be rated as complete when the patient is able to walk without support in the absence of pain or tenderness at the fracture site. Radiologically, the modified RUST score [18] will be determined by scoring each cortex on the anteroposterior (AP) and lateral radiographs as 1 = no callus, 2 = callus present, 3 = bridging callus, and 4 = remodeled, fracture not visible. The modified RUST score is the sum of these and therefore has a value from 4 to 16.

Malalignment, malrotation, and malunion will be determined by the treating surgeon. *Malalignment* will be measured on AP and lateral radiographs using the method described by Freedman et al. [19]. An angulation of > 5° in any plane is regarded as malalignment. *Malrotation* will be measured clinically and/or radiologically, depending on the availability of rotational CT and the local standard of care. A rotational deviation of > 10° is regarded as malrotation [20]. *Malunion* denotes that fracture-healing results in deformity corresponding to one or a combination of the following: > 5° malalignment, > 10° of malrotation, and/or a shortening of > 1 cm [20]. *Time to full weight bearing* is defined as the time elapsed between definitive treatment and the treating surgeon allowing the patient to bear full weight on the injured leg with or without any support. *Global health*, *pain interference*, and *physical function* will be documented using the corresponding domains of the Patient-Reported Outcomes Measurement Information System (PROMIS®) questionnaires. PROMIS® was established in 2004 with funding from the National Institutes of Health (NIH) as one of the NIH Roadmap initiatives for Medical Research and provided standardized and validated measures [21].

The questionnaires are designed to be completed by the patients without assistance. However, we anticipate that some patients may be unable to attend the planned FU visit(s). In these cases, patients will be contacted by e-mail or phone to document the outcome per interview. Interviews have been shown to produce similar scores compared to those obtained after completion of the questionnaires by the patients themselves [22, 23].

Validated translations of PROMIS® are available in an increasing number of languages. For this registry, only sites for which validated language versions are available will be asked to complete the forms. As other translations become available, these will be implemented immediately after notification of the respective IRB/EC.

The pre-injury status concerning global health and physical function will be captured retrospectively at baseline and entered in the database. The *global health assessment* reflects an individual’s evaluation of their physical, mental, and social health. In this registry, a short form of 10 questions (v1.2) will be used. The *pain interference* questionnaire covers pain interference with physical, social, and recreational activities and will be documented with a short form of 8 questions (v1.0 – Pain Interference 8a). The *physical function (mobility)* questionnaire assesses the patients’ ability to perform various daily living activities, documented in a short form of 10 questions (v2.0 – Physical Function 10b). *Pain* will be documented on a numeric rating scale (NRS) from 0 to 10 for the injured leg, the knee of the injured leg, and the ankle of the injured leg. *Procedure- or implant-related adverse events*, i.e., complications, will only be documented for the injured tibia, but not for concomitant fractures in the fibular, malleolar, or other regions.

In addition to AEs entered via free text, some pre-defined complications will be captured. *Intraoperative pre-defined complications* include iatrogenic fracture, iatrogenic vessel damage, excessive bleeding, iatrogenic nerve damage, and intraoperative resuscitation. *Postoperative pre-defined complications* include compartment syndrome, FRI, nonunion, refracture/peri-implant fracture, secondary loss of reduction, secondary displacement or breakage of the fixation material, deep vein thrombosis, pulmonary embolism, ongoing pain medication, or inability to bear full weight 1 year after definitive treatment.

FRI will be diagnosed as defined in the recently published consensus definition [24] and subsequent update [25]. Additional contextual information will be collected for all AEs and comprises the start date of the event, its severity and seriousness, information about the related additional treatment(s), date of recovery, and the event’s outcome.

### Data collection and management

Data handling and protection will be conducted according to the ISO 14155 guidelines, ICH-GCP, and applicable regulations. An electronic case report form (eCRF) has been designed in REDCap Cloud (https://www.redcapcloud.com/) to accommodate the registry’s specific features.

The REDCap Cloud is browser-based, metadata-driven, state-of-the-art electronic data capture software. Access to the eCRF is password-protected, and specific functions are assigned. The eCRF is to be completed promptly after a patient’s visit (i.e., 14 days after the occurrence of a documentable event).

During the site initiation visit and prior to recruiting the first patient, the research team at each site underwent a defined training program that includes explanations on inclusion and exclusion criteria, registry procedures, how to use the eCRF, and general aspects of ISO 14155 and GCP. Monitoring visits are to be performed on-site or remotely (via a web conference) as frequently as required to guarantee the completeness and accuracy of the information in the eCRF. Source data and any other essential documents must be archived according to the study site’s legal requirements. Clinical study data (i.e., eCRF) and essential documents will be archived by the sponsor according to the legal requirements.

### Premature termination

Due to the nature of the registry, no stopping rules have been defined. All treatments will be per standard of care, and no additional or investigational medical device or medication will be administered during the investigation. Patient participation in the study may end prematurely for one of the following reasons:

- The patient withdraws informed consent
- Screening failure (patient not meeting the eligibility criteria)
- Investigator’s discretion (e.g., patient noncompliance with the registry plan)
- Sponsor’s decision
- Unknown/lost to FU
- Death

In the case of premature termination, detailed information explaining the circumstances leading to termination will be obtained and recorded in the eCRF.

### Reporting of adverse events

Due to the nature of the registry, immediate reporting of AEs and serious AEs to the local EC/IRB is not required, unless indicated otherwise by the local EC/IRB. The occurrence of AEs and severe AEs will be summarized in annual reports and submitted to the EC/IRB, as required.

### Statistical analyses

As the registry aims are mainly descriptive and exploratory, patient characteristics and outcomes recorded at the standard of care scheduled FU assessments will be presented using simple summary statistics. Categorical variables will be summarized using the frequency and percentage for each category. Continuous variables will be summarized using the mean, standard deviation, median, interquartile range, and the minimum and maximum values. Additionally, summary statistics will be presented and stratified to clinically relevant categories, including the type of treatment received. Complications will be reported at both the patient and AE levels. Multiple events of the same type will be combined for each patient. Upon calculating complication rates, the denominator will be the total population size, irrespective of dropouts during FU. All complication rates will be presented with 95% confidence intervals. In addition to overall rates, complications will be presented according to categories for various relevant parameters, such as “action taken” (none/operative/nonoperative) or “outcome” (in progress/without persistent damage/with persistent damage/death). Patients with more than one event of the same type will be presented according to the most severe category.

To identify risk factors and evaluate specific research questions, it is planned to perform further appropriate statistics (e.g., multivariable analyses) if the volume and quantity of the collected data allows this. Enrolled patients who withdraw from the study FU for any reason (withdrawal of consent, death, loss to FU, etc.) will be included in the analysis until the time at which they withdrew.

### Ethical considerations and dissemination

This is an observational study on tibia fractures, often incurred through high-energy trauma such as road traffic accidents. Consequently, vulnerable patients who require immediate and/or emergency treatment, present with impaired decision-making capacity (temporarily or permanently), or who are only able to give oral consent may be included. In these cases, surrogate consent (study protocol proof; page 29) will be obtained unless otherwise indicated by the local EC/IRB and the patient’s informed consent will be obtained as soon as possible.

This study has been registered in Clinical Trials.gov under registration number NCT03598530. Ethics approval for this study was granted by the local EC or the IRB from each of the currently participating sites prior to patient enrollment. The results of this study will be published in peer-reviewed journals and presented at different conferences. This study will be conducted following the ethical principles established in the Helsinki Declaration updated in 2008.

## Discussion

TSFs are common injuries that can be challenging to treat due to the broad spectrum of fracture patterns, often combined with soft-tissue injuries. Therefore, musculoskeletal complications (i.e., FRI, nonunion) remain common. These complications not only influence the patient outcome but also place a cost burden on total healthcare expenditure [26]. Although treatment strategies have improved over recent years, complication rates remain higher compared to those of other anatomical locations and even compared to those related to many other surgically-treated disease entities [5, 27]. A better understanding of the epidemiology and pathogenesis of these complications is therefore essential because it can lead to prevention rather than treatment strategies [9].

Although high-quality studies have addressed questions related to the outcome of TSFs [28, 29], essential knowledge gaps – specifically within the field of FRI – remain. Multiple surveys indeed demonstrate a wide variability in clinical practice related to the prevention of musculoskeletal complications, especially in open fractures [27, 30, 31]. Puetzler et al. stated that global heterogeneity exists among orthopedic trauma surgeons concerning measures taken to prevent FRI [27]. This is in line with the widespread opinion among orthopedic trauma surgeons that adequate prevention guidelines based on solid scientific evidence are currently lacking [27].

One method for gathering more knowledge is the implementation of a high-quality, international, population-based registry. During the past decades, interest in orthopedic registries has grown exponentially [32]. Wennergren et al. recently published results from the Swedish Fracture Register [33, 34], mainly describing epidemiology. To date, however, most registries still primarily focus on arthroplasty [35, 36]. Therefore, the AO Foundation developed F.RO.S.T. Prospective data collection in a structured and systematic manner through the implementation of a registry will allow physicians to assess the association between different treatment strategies and patient outcomes, including complications. An important difference between F.R.O.S.T. and previous studies is that the data collection is extremely detailed, ranging from information on systemic and local antimicrobial prophylaxis to implant materials and the use of standardized outcome measures (e.g.*, FRI consensus definition*, PROs). Therefore, F.R.O.S.T. could identify risk factors in more detail, thereby improving strategies to prevent musculoskeletal adverse events.

In conclusion, although musculoskeletal complications (e.g., FRI) related to TSFs have already been described in ancient medical literature, internationally accepted guidelines regarding preventive measures remain scarce. While the exact repercussions of this lack of guidelines on daily clinical practice are unknown, standardized treatment protocols for TSFs based on scientific evidence are essential to optimize the outcome. The main purpose of the F.R.O.S.T. registry is to evaluate the association between different treatment strategies and patient outcomes. It will address not only operation techniques and implant materials but also perioperative preventive measures. For the first time, data concerning systemic perioperative antibiotic prophylaxis, the influence of local antimicrobials, and timing of soft-tissue coverage will be collected at an international level and correlated with standardized outcome measures in a large prospective, multicenter, observational registry.

## Supplementary Information

**Additional file 1.** Study protocol proof of the Fracture-Related Outcome Study for operatively treated Tibia shaft fractures (F.R.O.S.T.).

## Data Availability

Data sharing is not applicable to this article, as no datasets are generated or analyzed in the current study.

## Abbreviations

F.R.O.S.T.: Fracture-Related Outcome Study for operatively treated Tibia shaft fractures
TSF: Tibial shaft fracture
FRI: Fracture-related infection
PRO: Patient-reported outcome
AE: Adverse event
OTA: Orthopaedic Trauma Association
AO: Arbeitsgemeinschaft für Osteosynthesefragen
ICF: Informed Consent Form
IRB: Institutional Review Board
EC: Ethics Committee
FU: Follow-up
NIH: National Institutes of Health
eCRF: Electronic Case Report Form
NRS: Pain numeric rating scale
PROMIS: Patient-Reported Outcomes Measurement Information System
