# Supplementary material for: Fracture-related outcome study for operatively treated tibia shaft fractures (F.R.O.S.T.): registry rationale and design
Source: BMC Musculoskelet Disord. 2021 Jan 9;22:57. doi: 10.1186/s12891-020-03930-x (PMC7797092; doi:10.1186/s12891-020-03930-x)
Supplement: Supplementary file 1 — Additional file 1. Study protocol proof of the Fracture-Related Outcome Study for operatively treated Tibia shaft fractures (F.R.O.S.T.). [file 12891_2020_3930_MOESM1_ESM.pdf]

FROST registry

Fracture-Related Outcome Study for operatively treated Tibia shaft fractures (FROST)

Registry Plan

File name: FROST_RegistryPlan_16AUG2018_v2.0.docx

Version and date: Version 2.0, January 8, 2019

Principal Coordinating Investigator

**Prof. Dr. Willem-Jan Metsemakers**	Department of Trauma Surgery<br>UZ Leuven, Belgium<br><br>☎ +32 16344277 / +32 1631325<br>🖷 +32 16 34 46 14<br>Email: willem-jan.metsemakers@uzleuven.be

Co-Principal Coordinating Investigator(s)

**Prof. Michael J. Raschke, Dr. med.**<br>Klinik für Unfall-, Hand und Wiederherstrellungschirurgie<br>UKM Münster, Germany	**Prof. Dr. Michael H. J. Verhofstad**<br>Department of Trauma Surgery<br>Erasmus MC, The Netherlands

Sponsor

**AO Documentation and Publishing Foundation Clinical Investigation and Documentation (AOCID)**	Clavadelerstrasse 8<br>7270 Davos Platz, Switzerland<br><br>☎ +41 (0) 81 414 22 01<br>🖷 +41 (0) 81 414 25 82<br>Email: aocid@aofoundation.org

Confidentiality statement

The information provided in this document is strictly confidential and is available for review to investigators, potential investigators and appropriate Ethics Committees (ECs) or Institutional Review Boards (IRBs). No disclosure should take place without written authorization from the sponsor, except to the extent necessary to obtain informed consent from potential patients.

Content:

1 Synopsis ... 6
2 General information/responsibilities ... 10
2.1 Responsibilities ... 10
2.2 Study sites and investigators ... 11
3 Background and literature review ... 11
3.1 Incidence of tibial shaft fractures ... 11
3.2 Classification ... 12
3.3 Diagnoses and management of tibial shaft fractures ... 12
3.4 Complications and outcomes ... 12
3.5 Current status of research in this area ... 12
4 Registry implementation ... 12
5 Objective and hypothesis ... 13
5.1 Objective(s) ... 13
5.2 Hypothesis ... 13
6 Study design ... 13
6.1 Registry population and patient enrollment (Module 1) ... 14
6.2 Registry procedures and follow-up period ... 14
6.3 Study treatments ... 17
7 Definitions of outcome measures and study variables ... 17
7.1 Demographics ... 17
7.2 Medical history and pre-treatment values ... 17
7.3 Fracture(s) and injury(s) details ... 19
7.4 Treatment details ... 22
7.5 Clinical outcomes ... 24
7.6 Patient-reported outcome ... 25
7.7 Procedure- or implant-related adverse events ... 26
8 Statistical planning ... 27
8.1 Hypotheses and sample size considerations ... 27
8.2 Statistical analysis ... 27
9 Risk analysis ... 28
9.1 Patient risk analysis ... 28
9.2 Investigators risk analysis ... 28
9.3 Summary and conclusion ... 28
10 Informed consent process ... 29

10.1 General process for obtaining informed consent ... 29
10.2 Special considerations for patients unable to provide consent prior treatment .. 29
11 Adverse event reporting ... 29
11.1 Definitions ... 30
11.2 Adverse event documentation ... 30
11.3 Adverse event reporting ... 30
11.4 Follow-up of adverse events ... 31
12 Data management ... 31
12.1 Data collection, source data, storage and archiving ... 31
12.2 Imaging data ... 31
12.3 Confidentiality ... 32
13 Study management and quality control ... 32
13.1 Contract Research Organization ... 32
13.2 Training and organization at the study site ... 32
13.3 Study monitoring ... 32
14 Data Safety Monitoring Board ... 33
15 Regulatory affairs ... 33
16 Patient insurance ... 33
17 Study reports and publication policy ... 33
17.1 Annual reports and Final Study Report ... 33
17.2 Publication ... 33
18 Termination criteria ... 33
19 Deviations from the Registry Plan ... 34
20 Amendments to the Registry Plan ... 34
21 Time schedule ... 34
22 Quality assurance ... 35
23 Authors ... 36
24 Reference list ... 37

Appendix 1:
Signature of Principal Investigators from each study site

Appendix 2:
Publication policy version 1.0: July 16, 2018

Abbreviations/glossary

AE/SAE	Adverse Event(s)/Serious Adverse Event(s)
AO	Arbeitsgemeinschaft für Osteosynthesefragen = Association for the Study of Internal Fixation (ASIF)
AOCID	AO Documentation and Publishing Foundation, Clinical Investigation and Documentation
CRF	Case Report Form
CRA	Clinical Research Associate
EC	Ethics Committee
eCRF	Electronic Case Report Form
EDC	Electronic Data Capture
FRI	Fracture-Related Infection
FSR	Final Study Report
FU(s)	Follow-up(s), e.g. follow-up visit(s), follow-up procedure(s)
GCP	Good Clinical Practice
ICF	Informed Consent Form
ICH	International Council for Harmonisation of Technical Requirements for Pharmaceuticals for Human Use
IRB	Institutional Review Board
ISF	Investigator Site File
ISO	International Organization for Standardization
MeSH	Medical Subject Heading
MV	Monitoring Visit
PCI	Principal Coordinating Investigator (= Principal Clinical Investigator)
PI	Principal Investigator
SAP	Statistical Analysis Plan
SC	Study Coordinator
SD	Standard Deviation
SI	Sub-Investigator
SCV	Site Close-out Visit
SOP	Standard Operating Procedure

Definitions

Baseline	Status pre-treatment
Discharge	Discharge from orthopedic/trauma department
Eligible	Patients who meet all the inclusion and none of the exclusion criteria
Enrolled	Patient who consented
Follow-up visit	FU; visit at pre-defined time periods after the treatment day
Follow-up population	Patients intended to be followed-up (ie, eligible patients who commenced treatment within the study)
Pre-injury	Status before the injury (retrospectively assessed data)
Treatment day	"Day 0"; day of definitive treatment, i.e. in case of staged-procedure day of definitive fracture fixation
Written informed consent	Legally binding signature on the informed consent form (ICF) (i.e. either the patient, legally authorized representative or consultee), whereas the person who signs the ICF<br>• has the ability to understand the content of the patient information<br>• has signed and dated the EC/IRB approved written informed consent

1 Synopsis

**Official title**	Fracture-Related Outcome Study for operatively treated Tibia shaft fractures (FROST)
**Short title**	FROST registry
**Sponsor**	AO Documentation and Publishing Foundation<br>Clinical Investigation and Documentation (AOCID)
**Registration**	NCT03598530
**Background and purpose**	Tibial fractures are among the most common long-bone injuries. Because of the specific anatomical features of the tibia, over 15% of these fractures are classified as open. They represent the most common open long-bone injuries and consequently complications e.g. fracture-related infection (FRI) and compromised fracture healing occur frequently. Despite advances in modern fracture care, treatment of tibial shaft fractures still remains a challenge even in the hands of experienced trauma surgeons.<br><br>There is a limited amount of evidence on how to treat and prevent these complications, that not only reduce the quality of life of patients, but also provoke an enormous burden on overall healthcare costs.<br><br>Therefore, an urgent need exists to generate better evidence to improve care for patients with tibial shaft fractures and consecutively to decrease the financial burden on health care systems.<br><br>This registry, where patients are treated as per standard of care, offers a unique opportunity to document treatment data on tibial shaft fractures prospectively and to build a database in a systematic way. Such data will be mined to evaluate relationships between treatment and outcome as well as to investigate risk factors associated with complications.
**Clinical condition**	Patients sustaining a tibial shaft fracture (AO type 42)
**Intervention/procedure investigated**	Primarily planned fracture fixation, using osteosynthesis, including single or multiple staged procedures (e.g. first ExFix later conversion to internal fixation).<br><br>Note: All treatments will remain the standard (routine) care procedures based on individual clinician's judgement and the patient characteristics. The registry does not dictate any specific treatment.
**Comparison**	Not applicable. Since this is a patient registry, there is no comparator. All surgical treatments will be documented.
**Research type**	Observational
**Research design**	International, prospective, multicenter case series (registry)

**Short description**	Approximately 1'000 patients presenting with tibial shaft fractures (AO type 42) will be enrolled prospectively in this registry. All patients will be treated and followed at 6 weeks, 6 months and 1 year postoperatively always following the local standard of care (routine) visit schedule. To ensure proper outcome documentation, it is foreseen to contact patients at least via telephone or e-mail. In case of complications or eventful healing, patients will be followed up further as per the local standard of care (routine) visit schedule until fracture treatment is considered as complete by the treating physician to a maximum of 36 months if required.<br><br>Data collection will include patient and fracture details, treatment details, functional, clinical and patient-reported outcomes and anticipated or procedure- and implant-related adverse events (i.e. complications) and their corresponding treatment. Radiographs taken as per standard of care will be collected and healing status will be evaluated by the treating physician. Further analyses of the radiographs may be performed if necessary for medical/scientific reasons depending on initial exploratory analyses of the registry data.
**Objective(s)**	• To increase our knowledge and evidence with respect to the epidemiology and treatment concepts of tibial shaft fractures worldwide by prospective data collection in a structured and systematic way via the implementation of a registry. Creating such database allows data-mining, to generate hypotheses for further studies and to gather information to assist the clinical decision-making process<br>• To identify risk factors and their relationship with the outcome and complications<br>• To detect trends in the treatment of tibial shaft fractures and their complications
**Hypothesis**	Given the nature of the registry, there is no formal hypothesis a priori. Data from this registry will help to gather further clinical evidence, to drive further hypotheses or to answer unforeseen questions. Additionally, if from a medical scientific point of view needed to answer clinical questions of high relevance, the registry provides the possibility to nest other trials within the registry for a specific subpopulation (e.g. a randomized clinical trial for a specific subgroup of the registry).
**Parameters/Variables**	• Demographic data<br>• Medical history and pre-treatment values<br>• Fracture(s) and injury(s) details<br>o Injury(s) details<br>o Fracture classification<br>o Soft-tissue damage classification<br>o Neurovascular system injury classification<br>o Concomitant fractures<br>• Treatment details<br>o General treatment details

	- Specific treatment details<br>- Perioperative antibiotic prophylaxis<br>- Surgical technique (plate, nail, external fixation)<br>- Soft-tissue management<br>• Clinical outcome<br>- Time to bone healing/union<br>- Malalignment, malrotation and malunion<br>- Time to full weight bearing<br>• Patient-reported outcome<br>- PROMIS: Pain interference<br>- PROMIS: Physical function (mobility)<br>- PROMIS: Global heath<br>• Procedure- or implant- related anticipated adverse events (i.e. complications)
**Statistical considerations and estimated enrollment**	This registry is exploratory in nature. Therefore there is no formal statistical hypothesis and no sample size calculation. There are not *a priori* termination criteria such as a defined number of patients; however, the registry may be stopped for any of the following reasons:<br><br>• The registry has fulfilled its purpose (e.g. it is possible to determine the most effective treatment)<br>• Poor quality of data collected preventing the registry to fulfill its purpose<br>• Loss of funding, staffing or any other support required to carry out the registry.<br><br>Approximately 1'000 tibial shaft fractures are expected to be included in the 3 years enrollment period. The registry may continue open for further data collection should it be proven to be feasible and successful (see below for details).<br><br>Initially the data will be analyzed with the use of simple summary statistics. Further on, depending on the volume and quality of the collected data, different statistical analyses will be applied. Exploratory analyses will be conducted to investigate relationships between the different treatment options and the outcomes. If it is needed and from a medical/scientific point of view it seems important to answer a question of high clinical relevance, other trials for a specific subgroup of patients can be nested in the registry. This gives the unique possibility to employ the same patient population which is enrolled for the registry already.
**Start of enrollment**	November 2018 (estimated)
**Last patient/last visit**	Data collection may continue (see details above).<br>November 2022 (estimated for the first 1'000 patients)
**Eligibility inclusion criteria**	• Age 18 years or older at the time of the injury<br>• Diagnosis of a primary tibial shaft fracture (fracture type 42 according to the AO/OTA Fracture and Dislocation

	Classification) that will be treated operatively as part of standard of care.<br>• Informed consent obtained, i.e.:<br>○ Ability of the patient or assigned representative to understand the content of the patient information/ICF<br>○ Signed and dated IRB/EC-approved written informed consent
**Eligibility exclusion criteria**	• Pathologic fracture caused by malignancy<br>• Participation in any other medical device or medicinal product study within the previous month that could influence the results of the present study<br>• Patients who are not able to provide independent written informed consent unless defined and IRB/IEC-approved procedures for consenting such vulnerable patients are in place
**Study sites**	A maximum of 15 clinics in different regions will be initially selected via open call. Should the enrollment rate be lower than expected, it is planned to increase the number of participating sites.
**Health authority**	Local Ethics Committees and Institutional Review Boards
**Pubmed Medical Subject Heading [MeSH] terms and keywords**	• Tibial shaft fracture<br>• Fracture fixation<br>• Surgical treatment<br>• Fracture reduction
**Monitoring and administration**	All sites will receive the required documentation and will be trained and initiated formally by the sponsor. Administrative support will be established for all study sites and quality control measures (i.e. monitoring visits and audits) will be implemented to ensure adequate data quality.<br><br>Regular reviews of the status of the registry will be performed. Annual reports will be reviewed by all the stakeholders (sponsor, participating sites and funding bodies) to take a decision on continue or stop the registry. In particular, it is planned to broaden the scope of the registry by including secondary (i.e. revision) procedures and/or other intramedullary coated nail datasets (i.e. enriching the FROST registry dataset with patients at risk for developing complications; and using as platform to evaluate Expert Tibial Nail [ETN] PROtect).
**Imaging**	Images and clinical pictures collected following the local standard (routine) of care will be collected for later detailed analyses.

2 General information/responsibilities

The International Council for Harmonisation for Good Clinical Practice (ICH-GCP) guidelines define the responsibilities of the sponsor, investigators, and Clinical Research Associate (CRA) in the context of clinical investigations and are applicable for this research project. Specific topics, such as the permission from the responsible Ethics Committee (EC) or Institutional Review Board (IRB), are defined separately in the Registry Plan (RP). The study sites undertake to guarantee correct and prompt implementation, documentation, and transmission of the study data to the monitor.

The Principal Investigator (PI) and research team are instructed and trained about the conduct of the study by AOCID prior to the start of enrollment in order to verify their capability to cope with the required quality standards and compliance with the relevant standard operating procedures (SOPs). The Investigator Site File (ISF) containing all relevant study documents (i.e. essential documents) is provided to each clinic and needs to be kept up to date during the study period.

In the context of the registry, the participating sites will return the completely documented cases. Completely documented means that all Follow-up (FU) visits have been carried out for the cases included in the registry and that these assessments are recorded on the electronic Case Report Forms (eCRFs) specially designed for this registry. The PI at each study site signs a corresponding investigator contract, where all relevant aspects (legal, financial, etc.) are specified.

2.1 Responsibilities

**Principal Coordinating Investigator**	**Prof. Dr. Willem-Jan Metsemakers**<br>Department of Trauma Surgery<br>UZ Leuven, Belgium<br>☏ +32 16344277 / +32 1631325<br>🖷 +32 16 34 46 14<br>Email: willem-jan.metsemakers@uzleuven.be
**Sponsor**	**AO Documentation and Publishing Foundation**<br>**Clinical Investigation and Documentation (AOCID)**<br>Clavadelerstrasse 8<br>7270 Davos Platz, Switzerland<br>☏ +41 (0) 81 414 25 01<br>🖷 +41 (0) 81 414 22 82
**Director AOCID**	**Martin Schuler, PhD**
**Project Manager Clinical Operations AOCID**	**Víctor Díaz, PhD PMP**<br>AO Clinical Investigation and Documentation (AOCID)<br>Clavaderlerstrasse 8<br>7270, Davos Platz, Switzerland<br>☏ +41 81 414 25 07<br>Email: victor.diaz@aofoundation.org
**Head Medical Affairs AOCID**	**Alexander Joeris, MD, MSc HEPM**
**Statistics and Methodology AOCID**	**Kathrin Espinoza, MSc**
**Regulatory Affairs AOCID**	**Ivo Schauwecker, MSc**

2.2 Study sites and investigators

Participating study sites and investigators are listed in a separate document. A maximum number of 15 sites will be selected to commence the registry and achieve the initial target sample size. The sites will be located in multiple regions/countries. Over the course of the registry, should it be proven feasible and valuable (see section 18 for details on termination criteria), more sites may be included.

3 Background and literature review

3.1 Incidence of tibial shaft fractures

The tibial shaft, also known as diaphysis, is the part of the bone limited by the two bone end segments and is divided into three different regions as shown in figure 1.

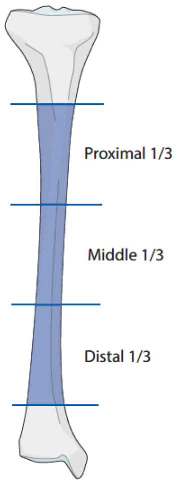

Figure 1. Tibial shaft and its three different regions.

Fractures of long bones constitute the majority of emergency operating room procedures in most trauma centers. Of these long bone injuries, tibial fractures are the most common. The incidence has been reported in several occasions and varies between 8.1 and 37.0 per 100'000 person-years (4, 9, 15). All reports consistently show a higher incidence in males compared to females regardless of the mechanism (high impact or low impact trauma) of injury. This incidence may have decreased over recent years according to a Swedish study between 1998 and 2004 (9).

The wide range of incidences reported in the literature can be attributed to regional differences, different time periods of data collection, or differences between studies in the access to entire population size. Overall, a more detailed overview of the incidence of tibial shaft fractures is not available, as for instance only two studies used a validated classification (4, 15). In addition, the incidence of associated fractures in the fibula or the malleolar region is not well known despite the impact of such fractures in the election of final treatment.

3.2 Classification

Correct classification of the fracture(s) is an essential step to the clinical decision-making process and to compare treatments and outcomes. In this registry the revised AO/OTA Fracture and Dislocation Classification (16), the Tscherne classification for closed fractures (1), the Gustilo (2) and the Orthopedic Trauma Association (12) classifications for open fractures, and the AO Grading System for neurovascular damage will be used to classify fractures and soft tissue injuries. The classifications and grading system are detailed below in section 7.

3.3 Diagnoses and management of tibial shaft fractures

As low- and high-energy mechanisms may be responsible, the clinical presentation may range from swelling of the lower leg, pain and an inability to weight bare, to complex open fractures with gross deformity. Diagnosis of the injury is made by a thorough physical examination (e.g. neurovascular status, compartment syndrome, open wounds), followed by plain x-ray (of the whole lower leg including the knee and ankle joints).

The management of tibial shaft fractures depends on the severity of the injury. Closed, nondisplaced fractures can, for example, be treated nonoperatively. Unstable (displaced) fractures where the axis cannot be maintained and fractures with concomitant soft tissue injuries should be treated surgically. The choice of the treating surgeon depends on the preferences and experience of the treating surgeon (11). Definitive surgical treatment options are: intramedullary nailing, plate osteosynthesis or external fixation (e.g. ring fixation). In cases where the patients' general condition (i.e. polytrauma) and/or the local soft tissue status (e.g. complex monotrauma; severe open fractures) don't allow for an immediate definitive treatment, a staged procedure can be necessary (i.e. primary external fixation).

3.4 Complications and outcomes

Because of the specific anatomical features of the tibia, over 15% of these fractures are classified as open, representing the most common open long-bone injuries (5, 13). This is one of the reasons why Fracture-Related Infection (FRI) and compromised fracture healing after treatment remain important complications. The FRI rate can range from 1% after operative fixation of closed low-energy fractures up to 25-30% in complex open tibia fractures (13). Nonunion rates can be up to 17% according to data from large teaching centers (6) and these numbers are higher when open fractures are involved (8). Overall, annual reoperation rates after the operative treatment of tibial shaft fractures have been reported to be between 12% and 44% (7).

3.5 Current status of research in this area

As previous data on complication and reoperation rates prove, despite advances in modern fracture care, tibial shaft fractures still remain a challenge even in the hands of experienced trauma surgeons.

There is a limited amount of evidence on how to prevent and treat these complications, that not only reduce the quality of life of patients, but also provoke an enormous burden on overall healthcare costs.

4 Registry implementation

It is envisioned that different modules are implemented in the registry and therefore the registry developed progressively. Depending on available funding and assuming the registry is proven to be feasible and successful, the registry will remain open without termination date (see specific termination criteria in section 18).

The following stage are thought to be implemented over time:

- **Module 1:** Prospective collection of treatment and outcome data on tibial shaft fractures excluding re-operation cases.
- **Module 2.1:** Enrichment of registry database by collecting additional retrospective data on tibial shaft fractures treated using the Expert Tibial Nail (ETN) with Protect Coating.
- **Module 2.2:** Enrichment of registry database by collecting additional retrospective and prospective data on re-operations/revision cases.
- **Module 3:** Nest studies and/or trials in specific subgroup(s) of patients if applicable/needed.

The current registry plan and protocol pertains to the implementation of module 1 only. Implementations of modules 2 and 3 MUST be notified and approved by the corresponding local EC/IRB.

5 Objective and hypothesis

5.1 Objective(s)

The registry's main objective is to collect data on the operative treatment and outcome of tibial shaft fractures in patients older than 18 years of age in an international and multicentre setting.

More in detail, this registry aims:

- To increase our knowledge and evidence with respect to the epidemiology and treatment concepts of tibial shaft fractures worldwide by collecting prospective data collection in a structured and systematic way via the implementation of a registry. Creating such database allows data-mining, to generate hypotheses for further studies and to gather information to assist the clinical decision-making process
- To identify risk factors (e.g. comorbidities) and their relationship with the outcome and complications.
- To assess the association between different treatment strategies and patient-related outcomes as well as treatment related complications.

5.2 Hypothesis

Given the nature of the registry, there is no formal hypothesis a priori. Data from this registry will help to gather further clinical evidence, to drive further hypotheses or to answer unforeseen questions.

Additionally, if from a medical/scientific point of view it is needed to answer clinical questions of high relevance, the registry will provide the possibility to nest other trials within the registry for a specific subpopulation (e.g. a randomized clinical trial for a specific subgroup of the registry) as mentioned in section 4.

6 Study design

This study is a prospective, multicenter, observational registry.

Treatments will be performed as per standard care (routine), i.e. the registry plan does not influence the clinical decision-making procedure, nor materials or surgical/imaging techniques.

6.1 Registry population and patient enrollment (Module 1)

6.1.1 Inclusion criteria

- Age 18 years or older at the time of the injury
- Diagnosis of a primary tibial shaft fracture (fracture type 42 according to the AO/OTA Fracture and Dislocation Classification) that will be treated operatively as part of standard of care.
- Informed consent obtained, i.e.:
    - Ability of the patient or assigned representative to understand the content of the patient information/ICF
    - Signed and dated IRB/EC-approved written informed consent

6.1.2 Exclusion criteria

- Pathologic fracture caused by malignancy
- Participation in any other medical device or medicinal product study within the previous month that could influence the results of the present study
- Patients who are not able to provide independent written informed consent unless defined and IRB/IEC-approved procedures for consenting such vulnerable patients are in place

6.1.3 Enrollment of patients

The participating study sites will identify all eligible patients (i.e. all patients who meet all inclusion and none of the exclusion criteria). The assessment of eligibility will be performed by the investigator and/or adequately trained member(s) of the clinical/research team. The assessment of eligibility may include an inquiry about the interest and willingness of the patient to participate in the research project. Data collection for this registry is not allowed without the consent of the patient, and therefore no data are collected in the eCRF prior obtaining consent by the patient as described in section 10 below.

All consented patients will be allocated to a unique patient number consisting of a combination of three letter and three numbers [XXX-000]. Each site will keep an Identification List linking the patient number with his/her personal information. Such Identification List is kept safe and in a locked place always. **Sites are not allowed to share the Identification List with any third party**. Sponsor representative, legal authorities, EC/IRB may have access to the Identification List during monitoring/auditing activities performed on-site.

For patients found to be ineligible after treatment, a protocol deviation will be documented indicating which in-/exclusion criterium (or criteria) was (or were) violated and when the protocol deviation was detected. The Sponsor will take the necessary actions as described in section 19.

The recruitment period is planned to elapse over 36 months to enroll approximately 1000 patients.

6.2 Registry procedures and follow-up period

The schedule of all FU visits as well as the data to be collected at each visit is shown in table 1.

Table 1: Registry schedule.

	Baseline	Treatment	Post-treatment visit 1[1]	Post-treatment visit 2[1]	Post-treatment visit 3[1]	Additional Post-treatment visit(s) [1]
	-	day 0	6 weeks (target 42 days: range 14 to 105)	6 months (target 183 days; range 106 to 260)	12 months (target 365 days, range 261 to 456)	Up to 36 months
**Eligibility**	X					
**Patient information/consent**	X[2]					
**Demographics**	X					
**Medical history and pre-treatment values**	X					
**Injury(s) details**	X					
**Treatment details**		X[3]				
**Clinical outcome(s)**			X	X	X	X
**Patient-reported outcome(s)**						
**PROMIS: Global Health**	X		X	X	X	X
**PROMIS: Physical Function**	X		X	X	X	X
**PROMIS: Pain Interferance**			X	X	X	X
**Pain NRS**			X	X	X	X
**Complications**			X	X	X	X
**Images and/or other clinical pictures**	X[4]	X[4]	X[4]	X[4]	X[4]	X[4]

1. In case a patient does not come for a visit in the specified time frame, the visits will be assigned according to the specified rules. Additional post-operative visits may take place up to 36 months after day 0.
2. Informed consent may occur after treatment under certain circumstances (see details in section 10). No data can be collected in the eCRF prior consent.
3. Treatment may occur the same day as the baseline. Treatment of tibial fractures may require two or more stages procedure (e.g. External Fixation and secondary internal fixation) performed in different days.
4. Images (X-rays, CTs, etc.) and clinical pictures are collected ONLY if they are taken as part of the standard of care (routine) procedures. All images and pictures are de-identified (see details in section 12).

All post-treatment visits with the defined time windows (Table 1) are calculated from the day 0 (i.e. the day of the definitive fracture fixation). Additional post-treatment visits may occur at any time (e.g. at three months) according to the local standard (routine) visit schedule. In particular, additional post-treatment visits after one year are expected in case of eventful healing and until the treating physician considers treatment as completed up to a maximum of 36 months.

To ensure that treatment can be regarded as completed, if a patient does not attend a visit (especially the last planned post-treatment visit at about 12 months), the research team may contact the patient via telephone or e-mail to collect outcome using the interview method and to ensure that no new complications have occurred.

6.2.1 Baseline visit

- All inclusion and exclusion criteria are checked to decide if the patient can participate in the study. Eligible patients are consented following local EC/IRB policies (see details in section 10).

- Consented patients are listed in the Enrollment Log maintained at each study site.
- Demographic data, injury details and medical history and pre-treatment values are documented.
- Images taken as per local standard of care (routine) procedures, are de-identified and transferred to AOCID via a secured server (e.g. sFTP).

6.2.2 Treatment visit (day 0)

- Patient is treated according to the decision of the treating physician following standard of care (routine) procedures. All details about the treatment are documented.

6.2.3 Post-treatment visits

- Clinical outcome and patient-reported outcome is documented.
- Details about complications and how they are treated are documented.
- Images taken as per local standard of care (routine) procedures, are de-identified and transferred to AOCID via a secured server (e.g. sFTP).

Post-treatment visits will occur at defined time points following standard (routine) visit schedule at each site. Patients will be contacted by e-mail or phone should they not attend the last follow-up visit to document the outcome (especially PRO) and to ensure no new complications have occurred. In case a patient does not come for a visit within the specified time frame, the visits will be assigned according to the specified rules.

Post-treatment visits will occur until up to 36 months follow-up period or, in cases of compromised or complicated healing, after the treating physician considers treatment as completed.

6.2.4 Unscheduled visits

Unscheduled visits can take place at any time during the study if a medical emergency occurs. Unscheduled visits are not *per se* documented in this study. Treatment- or condition-related Adverse Events (AEs) (i.e. complications) and their corresponding treatment, and dropout(s) are documented in specific forms within the eCRF.

6.2.5 Premature study termination

Patient participation in the study may end prematurely for one of the following reasons:

- Patient withdrew informed consent
- Screening failure (patient not meeting eligibility criteria)
- Investigator's discretion (e.g. patient noncompliance with RP)
- Sponsor's decision
- Unknown/lost to FU
- Death

For each case of premature termination, detailed information will be obtained explaining the circumstances leading to the termination. This will be recorded on an appropriate form in the eCRF.

If patients are withdrawn from the study, any collected data will be censored (i.e. data collected up to the time if withdrawn will be accounted for in the analysis).

6.3 Study treatments

This is a pure observational registry and all treatments are performed as per local standard (routine) of care. The registry plan does not influence the clinical decision-making procedure or materials and surgical/imaging techniques used.

The operative treatment for tibial shaft fractures in adults depend, among others, on the characteristics of the fracture or the extent of the soft tissue damage and can be performed in one or several stages (e.g. primary external fixation followed by later internal fixation). In this registry, details about all operative treatments are collected in the eCRF for further analyses.

Tibial shaft fractures may be accompanied with concomitant fractures of the proximal/distal tibial fibula and/or malleolar region. These fractures can be treated using lag screw (+/- washer) only, lag screw with or through buttress plate, lag screw and neutralization plate, antiglide plate, joint-bridging modular or triangular external fixation, and ORIF. Treatment details of these concomitant fractures will also be collected when applicable.

6.3.1 Allocation to study groups

The registry plan does not dictate any group allocation.

6.3.2 Postoperative care

There is no specific post-treatment care connected with the participation in the registry. Post-treatment care will be performed according to the standard of care (routine) procedures at each site.

7 Definitions of outcome measures and study variables

7.1 Demographics

The following data will be collected to obtain basic patient-demographic data.

- Gender
- Year of birth
- Height
- Weight
- Ethnicity
- BMI

7.2 Medical history and pre-treatment values

The following parameters will be collected about the medical history and pre-treatment values:

- Myocardial infarction: Yes/No
- Treatment or hospitalization for heart failure: Yes/No
- Treatment or hospitalization of vascular disease: Yes/No
- Peripheral vascular disease: Yes/No
    - If Yes: PAOD grade (Fontaine classification)
- Cerebrovascular accident or transient ischemic disease: Yes/No
- Hemiplegia: Yes/No
- Asthma, chronic lung disease, chronic bronchitis or emphysema: Yes/No
    - If Yes: Specify (COPD/Emphysema/Continuous supplemental oxygen/CF/Other)
    - If COPD: Gold Classification (0 at risk / 1 mild / 2 moderate / 3 severe /4 very severe)

- Diabetes mellitus requiring treatment: Yes/No
  - If Yes: Type I or Type II
  - If Yes: Status (de novo [i.e. hyperglycemia at ED/controlled/uncontrolled)
  - If Yes: Organ damage from diabetes (Yes/No)
- Moderate or severe renal disease: Yes/No
- Chronic liver disease: Yes/No
  - If Yes: Type/grade of disease (Mild/Moderate/Severe)
  - If Yes: Liver Cirrhosis (Yes/No)
    - If yes: Child-Puig Scale (A/B/C)
- Gastric or peptic ulcers: Yes/No
- Cancer (other than basal cell skin cancer): Yes/No
  - If Yes: Type (Lymphoma/Leukemia/Solid Tumor/Metastatic solid tumor/Other)
- Cognitive impairment due to dementia such as Alzheimer disease or any other etiology: Yes/No
- Rheumatic or connective tissue disease: Yes/No
- HIV or AIDS: Yes/No
  - If Yes: Recent CD4 count
- Other immunocompromised status: Yes/No
  - If Yes: Specify (Post-transplant/Long-term corticosteroids use/Other)
- ASA score
  - ASA Physical status 1 – A normal healthy patient
  - ASA Physical status 2 – A patient with mild systemic disease
  - ASA Physical status 3 – A patient with severe systemic disease
  - ASA Physical status 4 – A patient with severe systemic disease that is a constant threat to life
  - ASA Physical status 5 – A moribund patient who is not expected to survive without the operation
  - ASA Physical status 6 – A declared brain-dead patient whose organs are being removed for donors purposes
- Hypertension: Yes/No
  - If Yes: Status (Controlled with medication/Uncontrolled with medication/Uncontrolled without medication)
- Current smoking habit: Pack(s) per year calculated from the number of years of smoking habit and the number of cigarettes per day
- Chronic[1] use of analgesics: Yes/No
  - If Yes: Specify (NSAIDs/opioids/Other)
  - If known: Details about the dosage and the duration
- Current or previous consumption of alcohol: Yes/No
  - If known: Chronic/other
- Current or previous consumption of other substances (psychoactive substances and illicit drugs): Yes/No
  - If known: Details about the substance and duration
- Other pre-injury medication: Antibiotics therapy/Osteoporosis medication/Corticosteroids medication/Analgesics

[1] In the context of this registry. Chronic medication use is defined as ≥ 30 days of prescription prior to fracture with ≥ 1 day afterwards (Bucheit *et al.* (2018) *Injury*, 49; 1266-1271.

7.3 Fracture(s) and injury(s) details

7.3.1 Injury(s) details

The following data will be collected to obtain details about the injury(s).

- Date of injury
- Side of the injury
- Fracture mechanisms (15)
    - High energy trauma (i.e. fall from >3m or fracture due to traffic accident at more than 30 km/h)
    - Low energy trauma (i.e. rest of the falls/accidents)
- Classification of the tibial shaft fracture and soft tissue injury(s) as applicable and specified in sections below
- Presence of additional fractures in another region of the tibia, fibula or the malleolar region: Yes/No
    - If Yes: Side of fracture(s)
    - If Yes: Fracture classification according to the AO/OTA Fracture and Dislocation Classification (16)
- Polytrauma: Yes/No
    - If Yes: ISS score

7.3.2 Classification of tibial shaft fractures

Tibial shaft fractures will be classified according to the renewed AO/OTA Fracture and Dislocation Classification (16) as follow:

Table 2. AO/OTA tibial shaft fracture classification.

**Simple, spiral fracture**<br>**42A1***<br>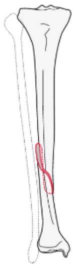	**Simple, oblique fracture**<br>**42A2***<br>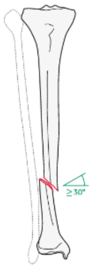	**Simple, transverse fracture**<br>**42A3***<br>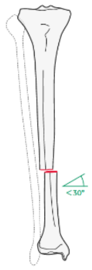
	**Intact wedge fracture**<br>**42B2***<br>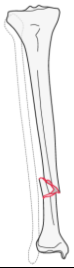	**Fragmentary wedge fracture**<br>**42B3***<br>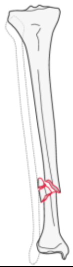
	**Intact segmental fracture**<br>**42C2**	**Fragmentary segmental fracture**<br>**42C3****

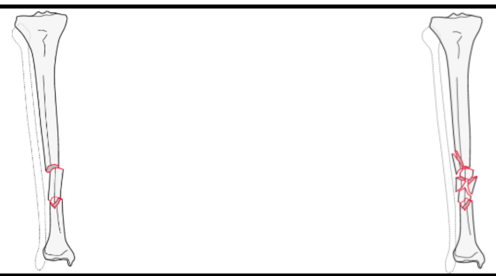

Asterisks define additional qualification (several qualifications can apply to the same fracture) as follow:
*	(a) proximal 1/3, (b) middle 1/3, (c) distal 1/3
**	(i) proximal diaphyseal-metaphyseal, (j) pure diaphyseal, (k) distal diaphyseal

7.3.3 Additional details for classification of closed fractures

For **closed fractures** the Tscherne's classification (1) will be documented as follow:

- **Closed fracture grade 0 (Fr. C 0):** There is no or minor soft-tissue injury with a simple fracture from indirect trauma.
- **Closed fracture grade I (Fr. C 1):** There is superficial abrasion or skin contusion, simple or medium severe fracture types.
- **Closed fracture grade II (Fr. C 2):** There are deep contaminated abrasions and localized skin or muscle contusions resulting from direct trauma. The imminent compartment syndrome also belongs to this group.
- **Closed fracture grade III (Fr. C 3):** There is extensive skin contusion, destruction of muscle or subcutaneous tissue avulsion (closed degloving). Manifest compartment syndrome and vascular injuries are included.

7.3.4 Additional details for classification of open fractures

For **open fractures** the Gustilo classification (2) and the Orthopedic Trauma Association (12) will be used. According to these classifications, the following information will be documented:

Type of fracture (Fig. 2)

- **Type I:** Fractures with a clean wound of less than 1 cm in size with little or no contamination.
- **Type II:** Skin laceration is longer than 1 cm but the surrounding tissues have minor or no signs of contusion.
- **Type IIIA:** There is still adequate soft-tissue coverage of the fractured bone, despite extensive soft-tissue laceration or flaps.
- **Type IIIB:** There is extensive soft-tissue loss with periosteal stripping and bone exposure.
- **Type IIIC:** Open fracture associated with arterial injury requiring repair.

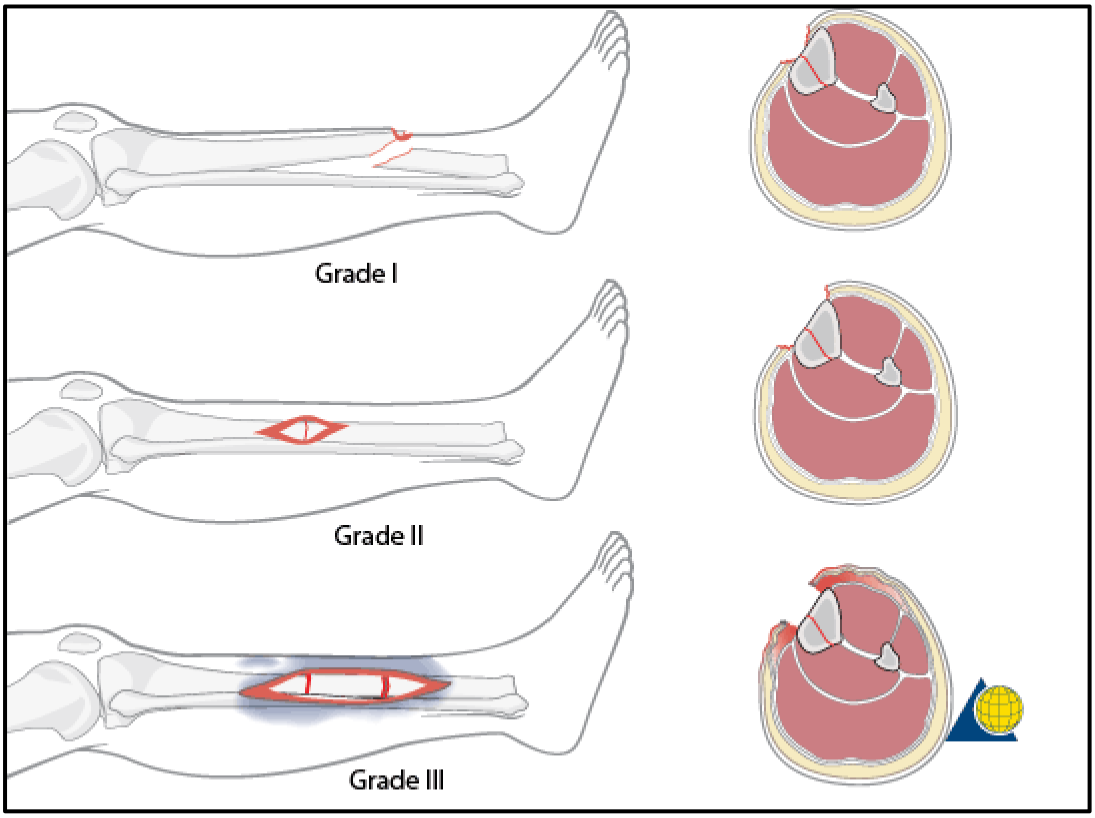

Figure 2. Summary of the three basic grades/types of open fractures.

Skin

- Can be approximated
- Cannot be approximated
- Extensive degloving

Muscle

- No muscle in area, no appreciable muscle necrosis, some muscle injury with intact muscle function
- Loss of muscle but the muscle remains functional, some localized necrosis in the zone of the injury that requires excision, intact muscle-tendon unit
- Dead muscle, loss of muscle function, partial or complete compartment excision, complete disruption of a muscle-tendon unit, muscle defect does not approximate

Arterial[2]

- No injury
- Artery injury without ischemia
- Artery injury with distal ischemia

Nerve[3]

- No injury
- Isolated nerve injury

[2] Details about the specific vessel damage will be documented, whenever possible.
[3] Details about the specific nerve damage will be documented, whenever possible.

Contamination

- None or minimal contamination
- Surface contamination (easily removed not embedded in bone or deep soft tissues)
- Imbedded in bone or deep soft tissues
- High risk environmental conditions (barnyard, fecal, dirty water, etc.)

Bone loss

- None
- Bone missing or devascularized but still some contact between proximal and distal fragments
- Segmental bone loss

7.3.5 Concomitant fractures

Tibial shaft fractures may be accompanied by other fractures. In the context of this registry, concomitant fractures in the proximal or distal segment of the tibia (AO fracture types 41 and 42), in the fibula (AO fractures type 4F) or in the malleolar region (AO fractures type 44) are of especial interest for their potential influence in outcome. Therefore, presence and detail classification according to the AO/OTA Fracture and Dislocation Classification system (16) of these fractures will be documented in the eCRF.

7.4 Treatment details

7.4.1 Tibial shaft fracture

Details about the treatment of the tibial shaft fracture will be collected in the eCRF following the schedule below. Additionally, other treatments resulting from the occurrence of complications will be documented in detail in appropriate forms of the eCRF.

7.4.2 General treatment details

- Surgical stages: One-stage / two- or multiple-stage procedure[4]
- Time from injury to index surgery (first procedure): in Hours
- Duration of surgery (skin-to-skin)
- Debridement: Yes/No
    - If Yes: Time elapsed between the accident and the debridement
    - If Yes: Irrigation type: Normal saline / Povidone-iodine / Hydrogen peroxide / Triclosan / Chlorhexidine / Polyhexanide / Castile soap / Antibiotic solution / Tap water / Other
    - If Yes: Irrigation pressure: High pressure (jet lavage; >20 psi) / Low-pressure (bulb syringe; 5 to 10 psi) / Very low pressure (gravity flow; 1 to 2 psi)
    - If Yes: Irrigation volume: 1-2 liters / 2-4 liters / 4-6 liters / 6-8 liters / 8-10 liters / >10 liters
- Skin preparation of surgical site (cleaning with water and soap) in addition to disinfection and sterile draping: Yes/No
- Estimated intra-op blood loss
- Application of tourniquet: Yes/No
- Local application of any product to promote bone healing: growth factors / other
- Reaming: Yes/No
    - If Yes: Largest diameter used

[4] In case of two- or multiple-stage procedures, details are collected for each stage as applicable.

- Surgical technique (reduction): Closed / mini open / extensive (full visualization of fracture site)
- Fixation method: Plate / Nail / External Fixation

7.4.3 Specific treatment details: Perioperative antibiotic prophylaxis

- Application of systemic antibiotic: Yes/No
    - If Yes: Specify type (e.g. cephalosporin)
    - If Yes: Additional coverage for gram negative organism (Yes/No) and time of removal
    - If Yes: Period (hours and/or days) of continuation of i.v. and/or oral antibiotics after fracture fixation
    - If Yes: Time of first administration: Several options
        - At the scene of the accident or during transport to the hospital
        - In the emergency department, as soon as possible upon arrival
        - Approx. 30 min before surgery, when narcotics are given
        - Postoperatively
    - If Yes: Adjustment of the dose to patients body weight: Yes/No
- Application of local antimicrobials: Yes/No
    - If yes: Several options
        - Antibiotic as powder (e.g. vancomycin)
        - Antibiotic impregnated collagen fleece/sponge
        - Subcutaneous infiltration of the wound with antibiotic fluid after wound closure
        - Antimicrobial wound dressings
        - Implant with antibiotic coating
        - PMMA Cement carrying antibiotics (e.g. gentamicin beads or cement spacer)
        - Antibiotic eluting bone void fillers for bone defects
        - Suture material with antimicrobial coating
        - Other
- Application of antibiotics (oral or systemically) for other reasons than prophylaxis (e.g. pneumonia, urinary tract infection, etc.)
    - If Yes: Specify

7.4.4 Specific treatment details: Plate

- Manufacturer
- Name of device
- Material
- Length (number of holes)
- Type of plate: Compression / Neutralization / Bridging
- For each type of implant: Manual bending (Yes/No)
- Screws (for each type of plate)
    - Type of screws: Standard cortical screws / Locking Screws / VA screws
    - Number of screws (per each type) placed proximal to the fracture and distal to the fracture
- Use of additional material(s): Cables / Bone graft and method (Reamer Irrigator Aspirator/Other) used / Other

7.4.5 Specific treatment details: Nail

- Manufacturer
- Name of device
- Material

- Length (mm)
- Diameter
- Approach: Suprapatellar / Infrapatellar / Parapatellar
    - If Infrapatellar: Through the patellar tendon / Medial to the patellar tendon
- Incision: Longitudinal / Transversal
- Number of dynamic interlocking screws (proximal and/or distal to the fracture)
- Number of static interlocking screws (proximal and/or distal to the fracture)
- Number of static Angular Stable Locking System (ASLS) proximal and distal to the fracture
- Use of end cap (Yes/No)
    - If Yes: Additional lengthening (Yes/No)
    - If Yes: Make most proximal screw angular stable (Yes/No)

7.4.6 Specific treatment details: External Fixation as definitive treatment

- Configuration: Modular external fixator / Uniplanar external fixation / Ring fixator
- Type: Acute shortening / Compression
- Diameter of the pins
- Number of pins proximal and distal to the fracture
- Manufacturer and name of device

7.4.7 Specific treatment details: Soft tissue management

- Primary closure of traumatic wound(s): Yes/No
    - If No: VAC: Yes/No
    - If No: Specify
- Delayed soft tissue coverage
    - If Yes: specification of kind of soft tissue coverage and time to coverage (in days) from the day of injury[5]
- Local flap: Yes/No
    - If Yes: specification of kind of flap and time to coverage (in days) from the day of injury
- Free vascularized flap: Yes/No
    - If Yes: specification of kind of flap and time to coverage (in days) from the day of injury

7.4.8 Concomitant fractures

Treatment of concomitant fractures in the fibula and/or malleolar region will be documented.

7.4.9 Additional treatments

Major additional treatments will be documented with Yes/No questions and without further detail.

7.5 Clinical outcomes

7.5.1 Time to bone healing/union

Time to bone healing is defined as the time elapsing between treatment and the bone union achievement. The treating surgeon will assess the healing status and healing progress based on the clinical appearance and the analysis of the available images (i.e. surgeons will be asked if bone union has been achieved). It will be a judgement of the treating surgeon taking the clinical context into account.

[5] If the exact number of days is unknow. It will be documented, whenever possible, the time to coverage/local flap/free vascularized flap as "within one week after injury" and "after one week of injury".

The healing status will be rated as either complete or incomplete, with “complete” being the combination of clinically, no pain or tenderness over the fracture site, and the patient able to walk without any means of support. Radiographically, surgeons will be asked to evaluate the available anteroposterior and lateral images to provide for each cortex (anterior, posterior, lateral and medial) and assessment as follow:

- Fracture line, no callus
- Fracture line, visible callus
- No fracture line, bridging callus
- No fracture line, remodeled

7.5.2 Malalignment, malrotation and malunion

Malalignment will be measured by the treating physician following methods published elsewhere (3). Briefly, varus-valgus alignment (i.e. lateral alignment) will be measured in AP radiograph as the angle between lines drawn perpendicular to and bisecting the tibial plateau and proximal medullary canal with a line bisecting the distal medullary canal and tibia plafond. Anteroposterior alignment will be determined on lateral radiographs by the angle between a line parallel to the proximal fragment and a line parallel to the distal fragment. An angulation >5 degrees in any plane will be considered malalignment.

Malrotation will be measured clinically or radiological (method of measurement will depend on the availability of a rotational CT and this was done as standard of care) and will be documented. An angulation >10 degrees will be considered malrotation (10).

Malunion will be defined as >5 degrees varus-valgus, >10 degrees malrotation, and >1cm shortening (10).

7.5.3 Time to full weight bearing

Time to full weight bearing will be defined as the time elapsing between the definitive treatment and the time at which the patient is allowed to be able to full weight bearing with or without other ipsilateral.

7.6 Patient-reported outcome

7.6.1 PROMIS

Patient-reported outcome(s) (PRO) will be documented at each FU visit as described in Table 1 above. More in detail, PROMIS (Patient-Reported Outcomes Measurement Information System) will be used to address pain interference and physical function. PROMIS forms are intended to be completed by the patient without help from anyone else; however, this is not always possible, and PROMIS measures have produced similar scores when the method of administration varied (e.g. self vs. interviewed).

- **Global health:** PROMIS global health assess an individual’s physical, mental, and social health. This score is intended to globally reflect individuals’ assessment of their health. In this registry a short form of 10 questions (v1.2); validated in Afrikaans, Czech, Danish, Dutch, English, Finnish, French, German, Italian, Japanese, Korean, Portuguese (Brazil), Simplified Chinese (Mandarin), Slovak, Spanish and Welsh, will be documented. To assess pre-injury status, patients will be asked to complete the score referring to their condition prior the injury.
- **Pain interference:** PROMIS pain interference assess self-reported consequences of pain on relevant aspects of one's life, including the extent to which pain hinders engagement in

physical, social or recreational activities. A short form of 8 questions (v1.0 – Pain Interference 8a) currently validated in Dutch, English, German, Hungarian, Portuguese and Spanish will be used in this registry.

- **Physical function (mobility):** PROMIS physical function assess self-reported capability rather than performance of physical activities including instrumental activities of daily living. In this registry a short form of 10 questions (v2.0 – Physical Function 10b) currently validated in Danish, Dutch, English, Finnish, German, Portuguese and Spanish will be used. To assess pre-injury status, patients will be asked to complete the score referring to their condition prior the injury.

Both instruments will be used only in sites where there is a validated language translation available. If new translations will become available over the course of the registry, these will be implemented immediately after notification to the corresponding EC/IRB.

Patients will be contacted by e-mail or phone should they not attend the follow-up visit(s) to document the outcome using the interview mode.

7.6.2 Pain NRS

Perception of pain will be assessed using a Numeric Rating Scale (NRS) from 1-10 where patient reports pain in the leg (e.g. knee and ankle) due to their tibia fracture.

7.7 Procedure- or implant-related adverse events

In this registry, only procedure- or implant-related adverse events (i.e. complications)[6] will be documented in the eCRF:

- **Intraoperative**
  - Iatrogenic fracture
  - Iatrogenic vessel damage/excessive bleeding
  - Iatrogenic nerve damage
  - Intraoperative resuscitation
- **Postoperative**
  - Fracture-Related infection (FRI): In the context of this registry, FRI will be defined following the recently published consensus definition (17). Two levels of certainty around diagnostic features were defined. Criteria could be confirmatory (infection definitely present) or suggestive. Four confirmatory criteria were defined: Fistula, sinus or wound breakdown; Purulent drainage from the wound or presence of pus during surgery; Phenotypically indistinguishable pathogens identified by culture from at least two separate deep tissue/implant specimens; Presence of microorganisms in deep tissue taken during an operative intervention, as confirmed by histopathological examination. Furthermore, a list of suggestive criteria has been defined.
  - Nonunion
  - Refracture/Peri-implant fracture
  - Compartment syndrome
  - Secondary loss of reduction
  - Secondary displacement of parts of the fixation material
  - Breakage of parts of the fixation material (e.g. autodynamization)
  - Deep Vein Thrombosis (DVT)
  - Pulmonary Embolism

[6] Complications will be documented only at the level of the injured tibia. Complications occurring due to the treatment of concomitant fractures in the fibular, malleolar region or other, will not be documented.

- Any other complication leading to a reoperation
- Inability to fully weight bearing at one year.
- Ongoing pain medication one year after treatment related to the fracture site

In the context of the registry, these events are considered as events of scientific interest, i.e. events that can clearly be connected to the treatment(s) or the medical condition under investigation. These events do not require immediate reporting unless they occur at a higher frequency and/or severity to that cited in the literature. A more detailed description of adverse event (AE) handling is provided in section 11.

8 Statistical planning

8.1 Hypotheses and sample size considerations

This registry is exploratory in nature. Therefore, there is no formal statistical hypothesis and no formal sample size calculation.

8.2 Statistical analysis

A detailed Statistical Analysis Plan (SAP) will be prepared for this study before the final analysis. An overview is presented in the following sections.

As the aim of this registry is mainly descriptive and exploratory, patient characteristics, and outcomes recorded at standard of care scheduled follow-up assessments will be presented using simple summary statistics. Categorical variables will be summarized using the frequency and percentage for each category. Continuous variables will be summarized using the mean, standard deviation, median, inter-quartile range, and minimum and maximum values. These summary statistics will in addition be presented according to clinically relevant categories, e.g. according to treatment received.

Complications will be reported both at the patient level and AE level. Multiple events of the same type will be combined for each patient. When calculating complication rates, the denominator will be the total population size, irrespective of dropouts during the course of follow-up; 95% confidence intervals will be provided for complication rates. For summaries of complications according to categories of severity or actions taken (no action/non-operative action/operative action), a patient with more than one event of the same type will be presented according to the most severe category or action.

Depending on the volume and the quality of the collected data as well as on the focus of specific research questions further appropriate statistical methods (e.g. multivariable analyses) will be applied.

8.2.1 Analysis populations

In general, all eligible patients who were treated within the registry will be included in the analysis. For specific research questions, only certain subgroups of patients may be relevant. In such a case, prior to the analysis it will be defined which patients will be included in the analysis.

8.2.2 Protocol deviations

Protocol deviations will technically include issues relating to i) eligibility and consent, ii) dropout from follow-up, and (iii) visits punctuality. The number and percentage of patients with a specific protocol violation will be presented and, if applicable, reasons for the violation will be listed.

8.2.3 Patient discontinuation

If a patient discontinues participation due to any reason, all data collected before discontinuation will be integrated into the analysis.

8.2.4 Missing data

Especially due to the registry design, some degree of missing data is expected for both baseline characteristics and outcomes. Patients may withdraw from the study prior to completion of the intended follow-up for reasons such as death, severe intercurrent illness, withdrawal of consent, and loss to follow-up. For each research question and each endpoint, a decision will be made as to whether special methods of analysis need to be employed to handle missing data. The decision will be based upon the extent of missing data, the likelihood of bias occurring as a result of missing data, and the importance of each endpoint.

9 Risk analysis

9.1 Patient risk analysis

9.1.1 Treatment-related risks

All images taken or treatments involved in this registry are widely used and considered as standard of care (routine) procedures. This registry plan does not dictate any specific treatment nor specific group allocation. Therefore, the participation in the registry does not pose additional treatment-related risk for the patient.

9.1.2 Registry-related risks

The main risk associated with the participation in the registry is loss of privacy. Patients' private and confidential medical information may get disclosed and confidentiality broken. See below for measures how to minimize such risks.

There are no other registry-related risks because the patients will be treated as per standard of (routine) care of the hospital.

9.1.3 Actions to minimize increased patients risks

General treatment-related risks will be present whether the patient participates in the study or not. Such risks will be managed by use of standards of treatment at the participating study sites. The risk of loss of privacy and confidentiality will be managed by strict adherence to data safety and security procedures explained elsewhere in this RP and in the applicable sponsor's SOPs.

The study will be implemented according to current valid international ICH-GCP guidelines and International Organization for Standardization (ISO) 14155. The ethical position is based on the Declaration of Helsinki, thus guaranteeing optimal protection of patient interests.

9.2 Investigators risk analysis

All treatments involved in this registry are widely used and considered as standard of (routine) care procedures. Therefore, the registry does not pose additional treatment-related risk for the investigators.

9.3 Summary and conclusion

This registry can be considered as a minimal risk study given that participation does not pose additional risk as compared with the standard of care (routine) procedures.

10 Informed consent process

10.1 General process for obtaining informed consent

Unless a waiver to obtain written informed consent is granted by the local EC/IRB, informed consent will be obtained before the treatment of the tibial fracture. However, if it is not possible to obtain consent prior to treatment (from the patient or surrogate; see below for details), patients may be consented after treatment given the pure observational nature of the registry.

In any case (unless a waiver from the local EC/IRB is granted), consent must be obtained before any data is collected in the eCRF.

The written patient information/ICF will explain benefits, risks, registry procedures and aim using a plain, understandable language. It will be explained to the patient that he/she is free to enrol in the registry and withdraw at any point without prejudice regarding the future treatment. The patient will signify his/her willingness to participate in the registry by signing and dating a personal copy of the approved ICF. The investigator will keep the original ICF in the ISF and provide the patient with a copy.

10.2 Special considerations for patients unable to provide consent prior treatment

Patients suffering from tibial fractures, especially those which production mechanism is a high energy trauma (e.g. road traffic accident), may be unable to provide consent prior treatment for some reasons as for example:

- Patient requires immediate and/or emergency treatment.
- Patient presents a temporal impaired decision-making capacity.
- Due to their injuries, patient is only able to give oral consent or assent.

In such cases, the following procedure to obtain surrogate consent will be implemented, should the corresponding EC/IRB not indicate otherwise.

- Surrogate consent will be obtained on behalf of the patient by an independent medical person (e.g. other doctor, nurse, etc.) not member of the research team, the patient's relatives or legal representative.
- The patient will be asked to provide consent for continued participation as soon as his/her medical condition allows it. If the patient disagrees to participate in the registry, the PI must ensure that no data is collected in the eCRF.
- If a patient remains unable to provide consent over the duration of the study, and if the initial surrogate consent was obtained from an independent medical person, the surrogate consent from a relative or legal representative must be obtained as soon as possible and prior any data is collected in the eCRF.

11 Adverse event reporting

In this registry, only adverse events/complications directly related to the condition or treatment of the tibial shaft fracture are collected. Based on the risk analyses and the observational nature of the study, AEs and Serious AEs (SAEs) are not reported to any data safety and monitoring board.

11.1 Definitions

11.1.1 Adverse event

An AE is defined as any untoward medical occurrence, unintended disease or injury, or untoward clinical signs (including abnormal laboratory findings) in patients, users or other persons whether or not they are related to the investigational medical device.

Note 1: This definition includes events related to the investigational medical device or the comparator.
Note 2: This definition includes events related to the procedures involved.
Note 3: For users or other persons, this definition is restricted to events related to investigational medical devices.

11.1.2 Serious adverse event

A SAE is defined as any AE that:

- led to death
- led to a serious deterioration in health of the patient that either resulted in
  - a life-threatening illness or injury, or
  - a permanent impairment of a body structure or a body function, or
  - inpatient or prolonged hospitalization, or
  - a medical or surgical intervention to prevent life-threatening illness or injury or permanent impairment to a body structure or a body function

OR

- led to fetal distress, fetal death or a congenital abnormality or birth defect

Note: Planned hospitalization for a pre-existing condition or a procedure required by the RP, without serious deterioration in health, is not considered as a SAE.

The definition of an AE does not imply that there is a relationship between the AE and the device or procedure under investigation.

11.2 Adverse event documentation

Occurrence of an AE/SAE will be documented in the appropriate eCRF form. The following information is collected:

- Name and description of the event
- Start date of the event (if applicable)
- Detailed information about the additional treatment(s) performed due to the occurrence of the AE
- Outcome of the event
- Date the patient has recovered from the event (if known)
- Severity of the event
- Seriousness of the event

11.3 Adverse event reporting

Unless it is indicated by the local EC/IRB, immediate reporting of AEs and SAEs to the local EC/IRB is not required in this registry. Occurrence of AE and SAEs will be summarized in annual reports and submitted to the EC/IRB as required.

11.4 Follow-up of adverse events

Each AE will be followed up until resolved with or without persistent damage or until the end of the patient's study participation, whatever occurs first.

12 Data management

12.1 Data collection, source data, storage and archiving

Data management will be performed by AOCID. Data handling and protection is conducted according to the ICH-GCP guidelines and applicable regulations.

A registry-specific Data Management Plan describes all details of data management and quality control of the FROST registry database at AOCID. The CRF collects data from each participating site. Specifically, all data on each patient enrolled in the study are documented in the CRF. The CRF contains data items as specified in this RP. Modification of the CRF will be made only if deemed necessary and in accordance with any amendment to the RP.

12.1.1 Electronic CRF

For this registry, an eCRF is designed to accommodate all the specific features. The electronic data capture system software solution used for this registry is specified in the Data Management Plan. Briefly, a browser-based, metadata-driven state-of-the-art Electronic Data Capture (EDC) software solution will be used.

Access to the eCRF is password protected and specific functions are assigned (e.g. SC, investigator, CRA, etc.). The eCRF is to be completed in a timely manner after a patient's visit (i.e. 14 days after occurrence of a documentable event).

After termination of the study, each study site will receive an electronic copy of the data collected at the respective study site.

12.1.2 Source data

Generally, data collected in the hospital patient charts are considered source data. In order to facilitate data collection at study sites, a set of data worksheets will be provided. On these worksheets, additional data may be collected that is not routinely documented in the hospital patient chart. For such additional data, the worksheet is considered to be the source document and has to be kept at the study site accordingly. Additional data that is not routinely documented in the hospital patient chart may be collected in the eCRF only. For such additional data, the data in the eCRF is considered to be the source data and there is no source document. Data collected on patient and surgeon questionnaires are also regarded as source data.

Source data and any other essential documents have to be archived according to legal requirements at the study site. Clinical study data reported in the eCRF and essential documents are to be archived by the sponsor according to legal requirements.

In case there are no other applicable law or policy at the participating site, the minimum archiving required by AOCID is 10 years after the Final Study Report (FSR) was finalized.

12.2 Imaging data

This registry plan does not dictate any specific image procedure neither for diagnosis or treatment. Each participating site will take images and clinical pictures according to the local standard of care

(routine) procedures. All images and clinical pictures taken will be de-identified and send to the Sponsor via secure File Transfer Protocol (sFTP) server.

Further details of image handling are provided in a separate image evaluation manual. Images will be stored in the database for later analyses, depending on the initial exploration of cases.

12.3 Confidentiality

Privacy and confidentiality of the patient's medical data will be maintained through the study. The eCRF and all other documents sent to the sponsor will be identified only with the numeric patient's identifier code. The study site will maintain the link between the patient identifier code and the patient's names.

Fully identifiable information may be reviewed for the purpose of verifying data in the eCRF only at the location of the study site. This can be carried out by the sponsor or sponsor’s designee, regulatory agencies or quality assurance personnel. Study site-specific regulations and procedures may apply and will be followed by the above listed personnel. Personal medical information will be treated as confidential always. The informed consent document will contain information about the confidentiality of the medical information and approval for the access.

13 Study management and quality control

13.1 Contract Research Organization

No Contract Research Organization (CRO) is contracted to perform this investigation.

13.2 Training and organization at the study site

Prior to recruiting the first patient, the registry site Principal Investigator (PI), Sub-Investigators (SI), and SC(s) will undergo a defined training program. This will occur during the site initiation visit. The training includes explanations of the registry procedures, inclusion and exclusion criteria, the eCRF and general aspects of ICH-GCP guidelines. The training will be performed by designated and adequately trained AOCID personnel. A training record is maintained for all registry site personnel involved in registry-specific activities. In the case of a change in the registry site personnel, the new staff must undergo the same training program.

The PI and the delegated personnel are responsible for managing the registry at each registry site. An ISF will be provided to each registry site for holding all essential documents and training documentation.

13.3 Study monitoring

A monitoring manual describes all monitoring procedures in detail.

Designated and adequately trained AOCID personnel (e.g. CRA) will perform on-site or remote (via web conference) monitoring visits (MV) as frequently as required. At these visits, the AOCID personnel will compare the data entered into the eCRF with the source data.

Direct access to source documentation must be allowed for the purpose of verifying that the data in the eCRF are consistent with the original source data. Findings from this review of eCRFs and source documents will be discussed with the study site personnel. AOCID expects that during monitoring visits, the relevant research team members will be available, the source documents will be accessible, and a suitable environment will be provided for review of all study-related documents. AOCID personnel will meet with the PI on a regular basis during the study to provide feedback on the study conduct.

14 Data Safety Monitoring Board

Since only standard of care (routine) procedures are performed in this registry and only anticipated condition-related adverse events are collected, it is not planned to set up a DSMB.

15 Regulatory affairs

The CIP, associated documents, investigator's financial disclosure, the patient information and ICFs will be sent to a respective EC/IRB for evaluation and approval. The relevant EC/IRB will be kept informed about the study progress and events according to their specific regulations and procedures.

If regulatory approvals are necessary in participating study sites, the study will commence in those places only after final approvals have been granted by the appropriate authorities. If required, the individual study site will have to submit the documents to any local review board as defined by the local hospital policy.

16 Patient insurance

As the patients are enrolled in an observational registry, no additional patient insurance will be obtained. Sponsor's general liability insurance is always valid and in full force.

17 Study reports and publication policy

17.1 Annual reports and Final Study Report

This registry is intended to remain open. Over the duration of the registry interim analyses will be reported in annual reports that would serve to take decision about implementation of subsequent steps (see section 4 for details). Annual reports will be submitted to the local EC/IRB as required.

The Sponsor will write a Final Study Report (FSR) with the support of the Principal Coordinating Investigators (PCIs) once the registry is closed. The FSR will be submitted to each local EC/IRB.

17.2 Publication

The publication strategy will be decided between the PCIs, AOCID and the Anti-Infection Task Force of the AO Foundation. Data will be available for publication to all investigators participating in the registry who will be allowed to request access to the multicenter data.

The procedure and review process to publish results from this registry are detailed in a separate publication guideline (see appendix 2).

18 Termination criteria

The progress of the registry, in particular the enrollment and the quality of the data collected, will be closely monitored by AOCID. There are no *a priori* termination criteria; however the registry may be stopped for any of the following reasons (14):

- The registry has fulfilled its purpose (i.e. it is possible to determine the most effective treatment)

- Poor quality of data collected preventing the registry to fulfill its purpose
- The registry is no longer relevant
- Loss of funding, staffing or any other support required to carry out the registry.

19 Deviations from the Registry Plan

Deviations from the procedures as described in this RP or altering the RP without following a defined process are not permitted. Should deviations from the protocol occur, AOCID must be informed in an expedited manner, depending on the seriousness of the deviation.

A RP deviation is any non-adherence to the protocol that does not involve the inclusion/exclusion criteria and GCP guidelines. RP deviations are minor and do not impact the study in a major way.

A RP violation is any significant divergence from the protocol on the part of the patient, investigator, AOCID, sponsor, or any other responsible party that affects e.g. the inclusion/exclusion criteria, GCP guidelines or patient safety and protection.

Deviations / violations will be recorded at the registry site and in the eCRF.

RP deviations and RP violations will be reported to the study sites and to the corresponding EC/IRB according to the applicable local policies and regulations. Corrective measures are taken depending on the significance and seriousness of the RP deviation. Such measures may be reminding the PI about his/her obligations, re-training personnel at the study site or, in serious cases, the closure of the study site and respective reporting to the EC/IRB. Serious cases of RP deviations include cases as listed above. Repeated violations may lead to closure of the study site.

20 Amendments to the Registry Plan

No changes to the approved RP are allowed, except when the change removes immediate threats for the patient safety or is of a purely administrative or logistic nature. Any change to the RP made to protect the life and well-being of the enrolled patients must be reported to AOCID within five days.

Should there be any need during the performance of the study to change this RP, an amendment will be developed, issued and approved by or notified to the required bodies as described in the AOCID internal procedures.

NOTE: Changes in the publication policy are not considered an amendment of the registry plan and do not require submission and approval by EC/IRB. AOCID will notify and agreed change in the publication policy to all participating sites.

21 Time schedule

Ethics Committee approvals	Sep 2018	to	Mar 2019
First patient/first visit			Nov 2018
Last patient/first visit			Nov 2022
Last patient/last visit			Dec 2023
Data analysis	Jan 2024	to	Jun 2024
Final Study Report	Jul 2024	to	Dec 2024

22 Quality assurance

AOCID's quality assurance policies and guidelines apply to the planning, selection of investigators and participating study sites, contracts, monitoring, recording of data and its analysis, documentation and archiving, evaluations, and reporting of the entire clinical investigation. The AOCID quality management system is based on the principles of ISO 9001 and ICH E6 Guideline.

23 Authors

CIP v1.0 developed by:

Prof. Dr. Willem-Jan Metsemakers	Department of Trauma Surgery, UZ Leuven, Belgium
Víctor Díaz, PhD PMP	Senior Project Manager Clinical Operations AOCID
Kathrin Espinoza, MSc	Project Manager Medical Statistics AOCID
Alexander Joeris, MD MSc HEPM	Head Medical Affairs and Health Economics

CIP v1.0 reviewed by:

Prof. Dr. Michael H. J. Verhofstad	Department of Trauma Surgery, Erasmus MC, The Netherlands
Prof. Michael J. Raschke, Dr. med.	Klinik für Unfall-, Hand und Wiederherstrellungschirurgie<br>UKM Münster, Germany
William T. Obremskey, MD, MPH, MMHC	Department of Orthopedic Surgery, Vanderbilt Medical Center, USA
Brigitte Gallo, PhD	Head Clinical Operations AOCID
Martin Schuler, PhD	Director AOCID

CIP v2.0 reviewed by:

Prof. Dr. Willem-Jan Metsemakers	Department of Trauma Surgery, UZ Leuven, Belgium
Alexander Joeris, MD MSc HEPM	Head Medical Affairs and Health Economics
Kathrin Espinoza, MSc	Project Manager Medical Statistics AOCID
Brigitte Gallo, PhD	Head Clinical Operations AOCID
Martin Schuler, PhD	Director AOCID

CIP approval signatures

Name	Function	Date	Signature
**Prof. Dr. Willem-Jan Metsemakers**	Principal Coordinating Investigator	10-01-2019	[signature]

Name	Function	Date	Signature
**Martin Schuler, PhD**	Director AOCID	8.01.2019	[signature]

24 Reference list

1. **Tscherne H, Oestern HJ**. A new classification of soft-tissue damage in open and closed fractures. *Unfallheilkunde*. 1982 3/1982;85(3):111-5.
2. **Gustilo RB, Mendoza RM, Williams DN**. Problems in the management of type III (severe) open fractures: a new classification of type III open fractures. *JTrauma*. 1984 8/1984;24(8):742-6.
3. **Freedman EL, Johnson EE**. Radiographic analysis of tibial fracture malalignment following intramedullary nailing. *ClinOrthop*. 1995 6/1995(315):25-33.
4. **Court-Brown CM, McBirnie J**. The epidemiology of tibial fractures. *J Bone Joint Surg Br*. 1995 May;77(3):417-21.
5. **Court-Brown CM, Rimmer S, Prakash U, et al.** The epidemiology of open long bone fractures. *Injury*. 1998 Sep;29(7):529-34.
6. **Coles CP, Gross M**. Closed tibial shaft fractures: management and treatment complications. A review of the prospective literature. *Can J Surg*. 2000 8/2000;43(4):256-62.
7. **Bhandari M, Guyatt GH, Tong D, et al.** Reamed versus nonreamed intramedullary nailing of lower extremity long bone fractures: a systematic overview and meta-analysis. *J Orthop Trauma*. 2000 Jan;14(1):2-9.
8. **Gaebler C, Berger U, Schandelmaier P, et al.** Rates and odds ratios for complications in closed and open tibial fractures treated with unreamed, small diameter tibial nails: a multicenter analysis of 467 cases. *J Orthop Trauma*. 2001 Aug;15(6):415-23.
9. **Weiss RJ, Montgomery SM, Ehlin A, et al.** Decreasing incidence of tibial shaft fractures between 1998 and 2004: information based on 10,627 Swedish inpatients. *Acta Orthop*. 2008 Aug;79(4):526-33.
10. **Investigators S, Bhandari M, Guyatt G, et al.** Study to prospectively evaluate reamed intramedually nails in patients with tibial fractures (S.P.R.I.N.T.): study rationale and design. *BMC Musculoskelet Disord*. 2008 Jun 23;9:91.
11. **de Boer P, Mogan SJ, van de Werken C**. *Orthopaedic Trauma Care*. Davos Platz, Switzerland: AO Publishing; 2009.
12. **Group OTAOFS**. A new classification scheme for open fractures. *J Orthop Trauma*. 2010 Aug;24(8):457-64.
13. **Papakostidis C, Kanakaris NK, Pretel J, et al.** Prevalence of complications of open tibial shaft fractures stratified as per the Gustilo-Anderson classification. *Injury*. 2011 Dec;42(12):1408-15.
14. **Gliklich R, Dreyer N, Leavy M**. *Registries for Evaluating Patient Outcomes: A User's Guide. (3rd edition)*. Rockville, MD: Agency for Healthcare Research and Quality; 2014.
15. **Larsen P, Elsoe R, Hansen SH, et al.** Incidence and epidemiology of tibial shaft fractures. *Injury*. 2015 Apr;46(4):746-50.
16. **Kellam JF, Meinberg EG, Agel J, et al.** Introduction: Fracture and Dislocation Classification Compendium—2018 International Comprehensive Classification of Fractures and Dislocations Committee. *Journal of Orthopaedic Trauma*. 2018;32:S1-S10.
17. **Metsemakers WJ, Morgenstern M, McNally MA, et al.** Fracture-related infection: A consensus on definition from an international expert group. *Injury*. 2018 Mar;49(3):505-10.

Appendix 1

Study site ____

RP approval signature of Principal Investigator

Name	Position	Date	Signature

With signing this statement, I agree and confirm:

- To have read and understood this RP and to have informed and to have supervised the appropriate training of all research team members of this study site involved with the conduct of the study.
- To assume responsibility to conduct the study in compliance with this protocol and future amendments at this study site.
- To obtain written approval from the IRB or the independent EC before initiating the clinical investigation at this study site.
- To not implement any changes to the protocol or the corresponding procedures without written agreement from the sponsor and the EC/IRB, except where necessary to eliminate immediate risk to the study patients.
- That I and all team members involved in the conduct of this clinical investigation are aware and trained in all relevant aspects of the ICH E6 (GCP) Guideline and all applicable regulatory requirements.
- That I and all team members involved in the conduct of this registry agree with the publication policy detailed in appendix 2

Appendix 2

PUBLICATION POLICY

Version 1.0: August 14, 2018

1. Purpose

The purpose of this guideline is to provide the principles that govern sharing and publication of data collected in FROST registry, in particular when data come from more than one participating site.

2. Scope

This guideline covers papers, abstracts and presentations that involve unpublished data collected within FROST registry. The approval of data use and sharing for publication purposes will be done by a guiding committee (hereafter called "FROST Registry Steering Committee"), whose tasks and responsibilities are described below. This guideline will remain in effect until further notice. Changes and modifications of this guideline must be agreed within the FROST registry Steering Committee and communicated to all participating sites in due time.

3. Right to access and use data

Any participating site will be able to use their **own data for non-commercial internal and educational purposes** (e.g. annual summary reporting, presentations at symposia or meetings) and publication of such data in thesis or dissertations. However, if a participating site wants to use its **own data to publish in a peer-reviewed journal, national and international conferences publishing abstracts in journals**, it will require approval by the FROST Registry Steering Committee according to the workflow described below.

Any participating site is also entitled to **access and use aggregated data** (i.e. data from several participating sites). Access and use of aggregated data requires approval by the FROST Registry Steering Committee and will be allowed after a minimum of 200 patients have been enrolled and completed the 1-year follow-up visit.

4. FROST Registry Steering Committee

4.1. Responsibilities

The goal of the FROST Registry Steering Committee is to oversee and regulate methodological, scientific and legal aspects related to AO registries. It makes sure that registries and data collected meet quality and regulatory standards, checks for compliance of the sites and regulates the use and sharing of data. It also promotes the accurate, scientifically sound and objective presentation of results from the FROST registry. It ensures that involved parties have consented to the use of their data, solves conflicts of interest and avoids duplication of efforts and data mining.

The FROST Registry Steering Committee reviews proposals, makes suggestions and advises the Anti-Infection Task Force and the TK Trauma to approve or decline projects for analysis and publication of i) own data aimed to be published in a peer-reviewed journal or national and international conferences publishing abstracts in journals; or ii) use aggregated data. In addition, the FROST Registry Steering Committee reviews the final abstract or publication and maintains a record of proposed and published papers and presentations from the FROST registry.

4.2. Composition

Members of the FROST Registry Steering Committee are the following:

- PCI
- Co-PCI(s)

- Anti-infection Task Force or other group as delegated by AOTK.
- Head of Medical Affairs and Health Economics AOCID or delegated person
- Medical Statistician AOCID
- Project Manager AOCID

5. Process and approval of projects for analysis and publication

5.1. Own data

To initiate the analysis and publication using their own data for non-commercial internal and educational purposes from FROST registry, investigators should request via e-mail to the Project Manager an export of the data. A copy of the final draft should be sent to the FROST Registry Steering Committee prior to publication. The FROST Registry Steering Committee will perform checks for compliance and ensure that funding is properly acknowledged. Applicants should receive a response within 2 to 4 weeks (depending on whether an abstract or full manuscript has to be reviewed).

To request their own data from the FROST registry for analysis and publication in a peer-reviewed journal or national and international conferences publishing abstracts in journals, investigators should submit a proposal (see appendix) to the FROST Registry Steering Committee. Applicants should receive a response within 2 to 4 weeks. If the FROST Registry Steering Committee evaluates the proposal positively, the investigators will receive an export of the data. A copy of the final draft should be sent to the FROST Registry Steering Committee prior to submission of the abstract/manuscript. The FROST Registry Steering Committee checks for compliance and ensures that funding is properly acknowledged within 2 to 4 weeks.

The flowchart below describes the process to access and use own data.

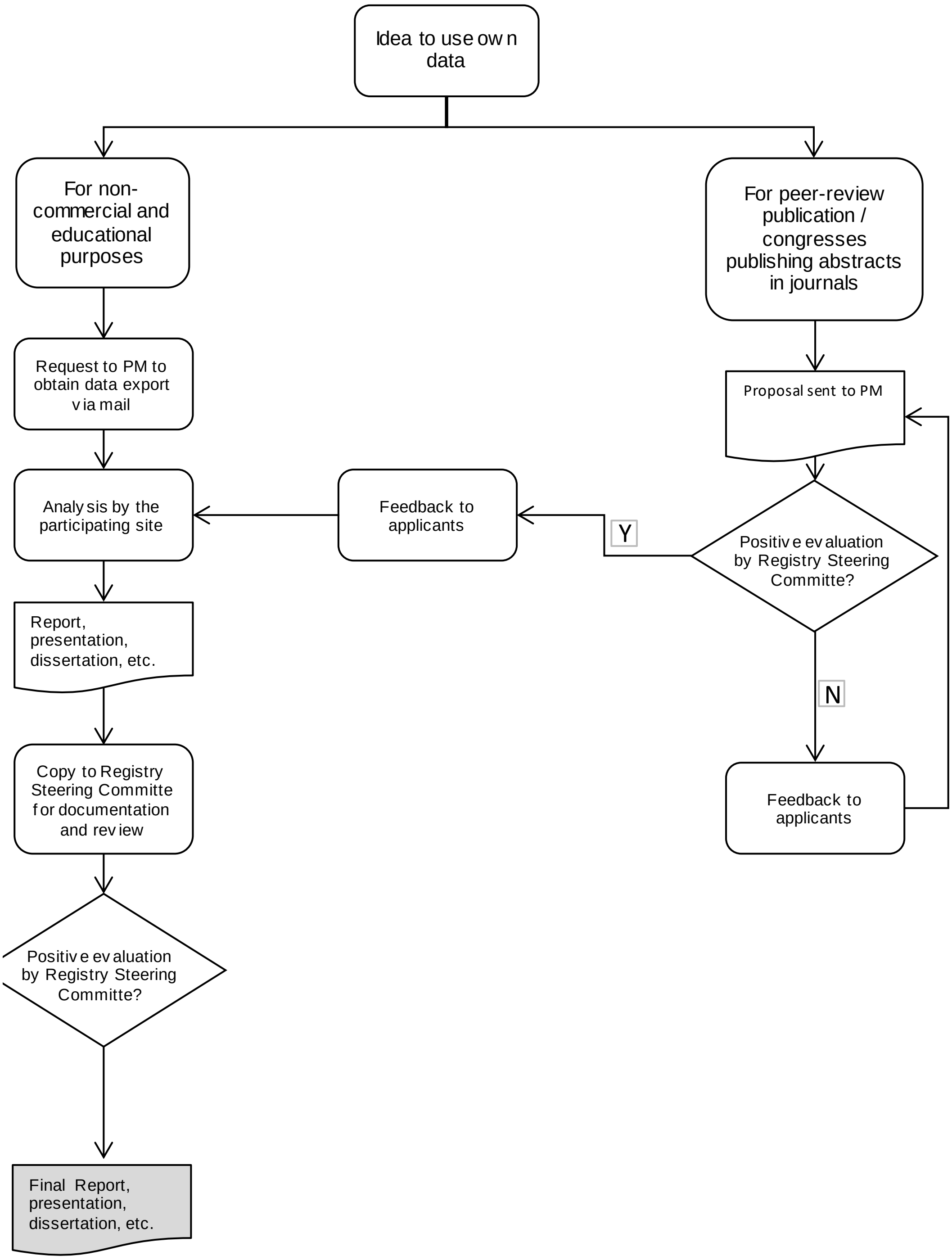
Idea to use own data
For non-commercial and educational purposes
For peer-review publication / congresses publishing abstracts in journals
Request to PM to obtain data export via mail
Proposal sent to PM
Analysis by the participating site
Feedback to applicants
Y
Positive evaluation by Registry Steering Committe?
Report, presentation, dissertation, etc.
N
Copy to Registry Steering Committe for documentation and review
Feedback to applicants
Positive evaluation by Registry Steering Committe?
Final Report, presentation, dissertation, etc.

5.2. Aggregated data

To request aggregated data from FROST registry for analysis and publication, investigators should submit a proposal to the FROST Registry Steering Committee. Applicants should receive a response within 2 to 4 weeks. If the FROST Registry Steering Committee evaluates the proposal positively, the final proposal and the budget will be submitted to the next AO TK meeting at least 3 weeks before it takes place (dates will be communicated regularly). If the proposal is accepted by the AO TK, AOCID will analyze the data and will provide the appropriate tables and figures. This ensures that the presentation of the results will be consistent through all FROST publications and will avoid duplications or conflicts among different papers. In addition, investigators will have the possibility to request support from AOCID's Medical Writing department to develop the entire manuscript. Should the investigators decide to prepare the manuscript without AOCID's support, approval of the FROST Registry Steering Committee prior to submission is mandatory to ensure scientifically sound and objective presentation of results as well as proper acknowledgement of the funding.

The flowchart below describes the process to access and use aggregated data.

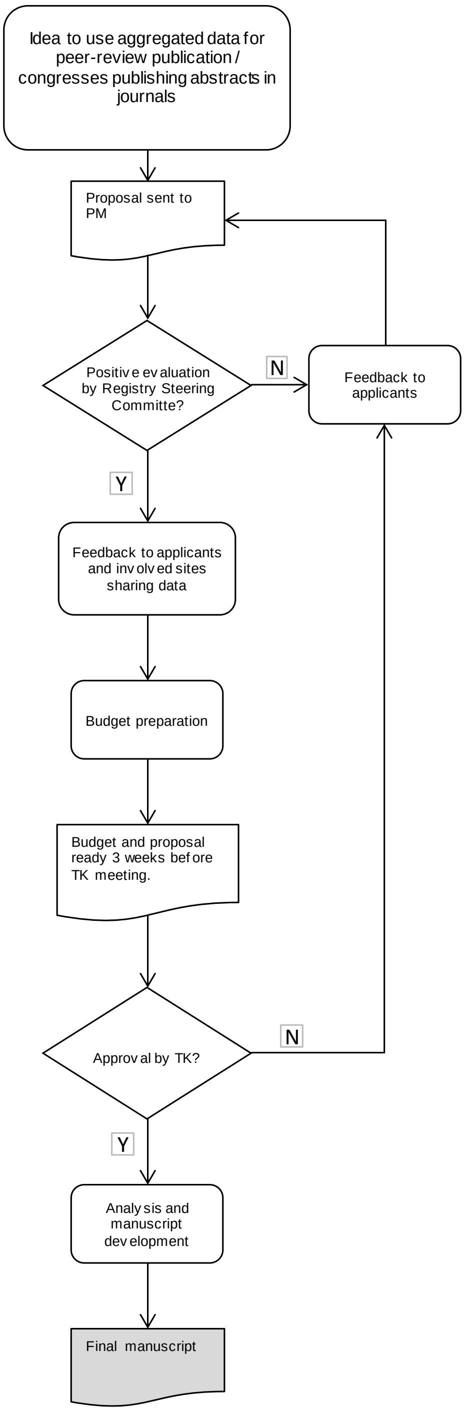

6. Authorship

The authorship of any publication, manuscripts, poster or oral presentations, or other reports of the results of FROST registry will be guided by the criteria formulated by the International Committee of Medical Journal Editors (http://www.icmje.org). Authors should meet the following criteria:

- Substantial contributions to the conception or design of the work; or the acquisition, analysis, or interpretation of data for the work; AND
- Drafting the work or revising it critically for important intellectual content; AND

- Final approval of the version to be published; AND
- Agreement to be accountable for all aspects of the work in ensuring that questions related to the accuracy or integrity of any part of the work are appropriately investigated and resolved.

All individuals who meet the first criterion should have the opportunity to draft, review and approve the version to be published. Due to the potential number of authors, it may not be realistic to expect that every single author contribute to draft the manuscript. Therefore, a core writing group (2-3 authors) may be established, while the rest of the authors will be asked to critically review and approve the final version. This should be specified in the initial proposal.

As a large multicenter registry, any publication resulting from aggregated data will aim for a journal without restrictions in the list of authors. A study group, named e.g. "FROST registry study group" should be implemented to prevent lists of numerous co-authors in publications. Each participating clinic should have the opportunity to propose author(s) for the study group, provided they meet the above mentioned ICMJE criteria. Should the journal limit the number of authors, the leading authors for the respective manuscript/abstract should be named. All other co-authors are included in the study group. For example: *Joe Don, Peter Smith, Tom Taylor and FROST registry research group (the names of the other members of the research group are listed separately in the manuscript and count for all listed authors as publication as well).*

The order of the authors will be defined in the initial proposal and approved by the FROST Registry Steering Committee.

Credit will be given to the other participants of the study such as associated staff at participating centers and will be listed according to the journal's guidelines.

7. Availability and analysis of data request by external investigators

The availability of the data to external investigators will depend on approval from the FROST Registry Steering Committee and will follow the process detailed in section 5.2. above.

8. Data request by DePuy Synthes (DPS)

DPS is entitled to make regular requests of aggregated data for internal use and to support regulatory submissions. The data will be disclosed publicly.

Request of aggregated data should be addressed to AOCID, who informs AO TK.

9. Data request by industry entities or their representatives other than DPS

In general, industry data use requests are those that originate from an industry entity or their representatives and are intended for their corporate activities (e.g. marketing, sales, business development, R&D, etc.).

Other manufacturers than DPS will be permitted to request access to their own implant-specific information from FROST data via “standardized” and custom Industry data reports. The reports will be subject to fair market value pricing for eligible industry purchasers.

Data Request Process: AOCID and AOTK will evaluate and potentially approve these requests. The AOCID and AOTK retain the right to refuse any request, to limit the data made available, and to limit the period of data use for any reason; particularly if there is concern for misuse.

Prior to disclosure, any data regarding other companies' implants will be de-identified and/or aggregated to prevent unfair exploitation of other companies' implant-specific data. For example, implant usage/performance reports may illustrate specific data for the requesting companies' implants versus category averages.

Request of FROST registry data for publication

**Type of application**	☐ Publication of own data in a peer-review journal<br>☐ National or international conference publishing abstracts in journals<br>☐ Publication of aggregated data
**Target journal**	
**If aggregated data, please specify**	☐ All sites ☐ Specific sites
**If specific sites, please list them**	
**Title**	
**Research Question**	
**Authors list. Planned authors taking into account the ICMJ criteria.**	Author 1: Name / Institution<br>Author 2: Name / Institution<br>Author 3: Name / Institution<br>Author n: Name / Institution
**Applicant**	Name:<br>Institution:<br>E-mail:<br>Phone number:
**Request Medical Writing support from AOCID**	☐ Yes<br>☐ No
**Comments**	
**Date and signature**	
